# Design, Synthesis and Anti-Tumor Activity of Novel Benzimidazole-Chalcone Hybrids as Non-Intercalative Topoisomerase II Catalytic Inhibitors

**DOI:** 10.3390/molecules25143180

**Published:** 2020-07-12

**Authors:** Wei Zhou, Wenjin Zhang, Yi Peng, Zhi-Hong Jiang, Lanyue Zhang, Zhiyun Du

**Affiliations:** 1 School of Biomedical and Pharmaceutical Sciences, Guangdong University of Technology, Guangzhou 510006, China; wenjinzhang8@163.com (W.Z.); doris_py@163.com (Y.P.); zhanglanyue@gdut.edu.cn (L.Z.); 2 State Key Laboratory of Quality Research in Chinese Medicine, Macau Institute for Applied Research in Medicine and Health, Macau University of Science and Technology, Avenida Wai Long, Taipa, Macau 999078, China; zhjiang@must.edu.mo

**Keywords:** type II topoisomerase, benzimidazole, chalcone, anti-tumor

## Abstract

Chemical diversification of type II topoisomerase (Topo II) inhibitors remains indispensable to extend their anti-tumor therapeutic values which are limited by their side effects. Herein, we designed and synthesized a novel series of benzimidazole-chalcone hybrids (BCHs). These BCHs showed good inhibitory effect in the Topo II mediated DNA relaxation assay and anti-proliferative effect in 4 tumor cell lines. **4d** and **4n** were the most potent, with IC_50_ values less than 5 μM, superior to etoposide. Mechanistic studies indicated that the BCHs functioned as non-intercalative Topo II catalytic inhibitors. Moreover, **4d** and **4n** demonstrated versatile properties against tumors, including inhibition on the colony formation and cell migration, and promotion of apoptosis of A549 cells. The structure-activity relationship and molecular docking analysis suggested possible contribution of the chalcone motif to the Topo II inhibitory and anti-proliferative potency. These results indicated that **4d** and **4n** could be promising lead compounds for further anti-tumor drug research.

## 1. Introduction

Type II topoisomerase (Topo II) is a predominant enzyme involved in DNA replication, recombination, and repair [1,2]. Due to its important roles in cell life cycle, Topo II has become a prominent target for anticancer drug research [3,4]. In the last decades, typical Topo II inhibitors, such as etoposide and doxorubicin, have been clinically applied as anti-cancer drugs. However, possible serious risk of drug resistance and side effects limits their wide therapeutic use [5,6]. Topo II inhibitors can be classified into Topo II poisons and Topo II catalytic inhibitors according to their different mechanism of action [7,8]. The former type stabilizes the covalent DNA-Topo II cleavable complexes while the latter type inhibits any other step in the catalytic cycle. Accumulating evidences have suggested that Topo II catalytic inhibitors may be more potent and less toxic than poisons [2]. Moreover, catalytic inhibitors could resist possible side effects of poisons [9,10]. Therefore, the research for novel Topo II catalytic inhibitor is of great significance.

**Figure 1:**
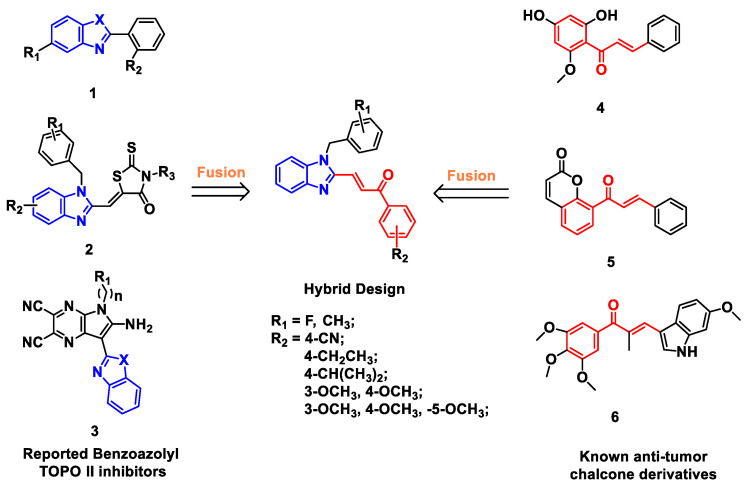
Design of benzimidazole-chalcone hybrids as topoisomerase II inhibitors.

Benzimidazoles have various of biological activities, such as anti-bacterial, anti-inflammatory, anti-tumor, and so on [11,12,13,14,15]. Studies have shown that the benzimidazole scaffold rings play a critical role in the antitumor activity [16,17]. In recent years, the modification of benzimidazole rings has become one of the hottest topics in the development of anti-cancer agents. Several benzimidazole derivatives have been reported as new Topo II inhibitors (**1**, **2** and **3,** Figure 1) [18,19]. Mechanistic and molecular docking studies have revealed that benzimidazole-containing derivatives act as Topo II catalytic inhibitors by blocking the adenosine triphosphate (ATP) binding site of the enzyme [16,18,20].

Chalcone derivatives have also been extensively investigated for their various physiological activities, such as anti-cancer, anti-oxidant, anti-inflammatory, anti-infective activities [21,22]. Naturally occurring or synthetic chalcone derivatives (**4**, **5** and **6**, Figure 1) have attracted much attention not only for their good tumor inhibitory activity, but also for their relatively safety for healthy tissues [23,24,25,26,27].

**Figure 2:**
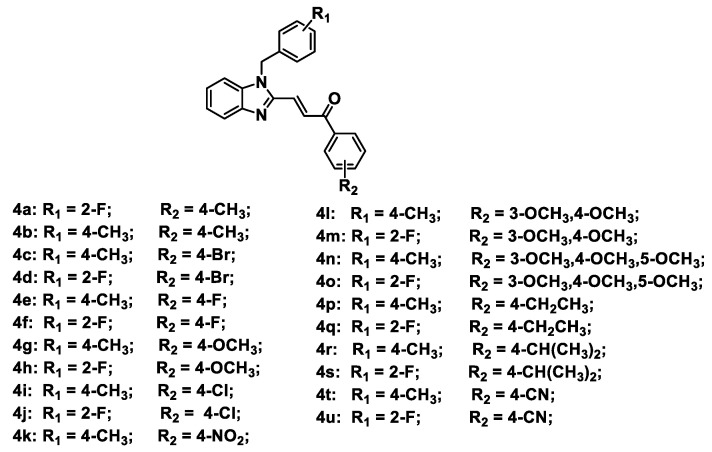
Chemical structures of designed Topo II inhibitors **4a**–**4u.**

Based on the facts above, we focused on Topo II inhibitors, and designed a series of benzimidazole derivatives cooperated with the chalcone fragment using a classic pharmacophore hybridization strategy (Figure 1 and Figure 2) [28,29]. Compounds were synthesized and evaluated for Topo II inhibitory activity, and their mechanism of action were also explored. The results indicated that benzimidazole-chalcone hybrids (BCHs) were a class of non-embedded Topo II catalytic inhibitors. Moreover, most of the BCHs were found to not only effectively inhibit catalytic function of Topo II, but also suppress cell proliferation (HepG2, A549, LNCaP and MG63) and induce cell apoptosis.

## 2. Results and Discussion

### 2.1. Chemistry

**Scheme 1:**
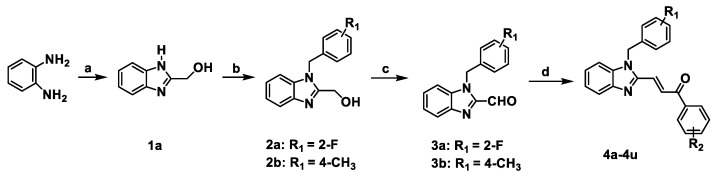
The synthetic route of **4a**–**4u**. *Reagents and conditions*: (**a**) glycolic acid, 4N HCl, 100 °C, 6 h; (**b**) benzyl bromides, K_2_CO_3_, DMF, rt, 24 h; (**c**) Dess-Martin periodinane, DCM, 0 °C, 1 h; (**d**) ArCOCH_3_, EtOH, 10% NaOH, rt, 1 h.

A series of benzimidazole-chalcone hybrids **4a**–**4u** was synthesized as described in Scheme 1. (1*H*-Benzo[*d*]imidazol-2-yl) methanol (**1a**) was obtained by refluxing *o*-phenylenediamine with glycolic acid in hydrochloric acid. The substituted-(1-benzyl-1*H*-benzo[*d*]imidazol-2-yl) methanols **2a**, **2b** were synthesized with 47-88% yields using **1a** and appropriate benzyl bromides in the presence of K_2_CO_3_. Subsequently, substituted-1-benzyl-1*H*-benzo[*d*]imidazole-2-carbaldehydes **3a**, **3b** were obtained in 56–78% yields by oxidation of the corresponding primary alcohols with Dess–Martin reagent. The target compounds **4a**–**4u** were finally prepared in 70–86% yields from **3a** or **3b** by using the Claisen-Schmidt reaction in the presence of appropriate acetophenones. Chemical structures of the target compounds **4a**–**4u** are presented in Figure 2**.**

### 2.2. BCHs as Potent Topo II Inhibitors

**Figure 3:**
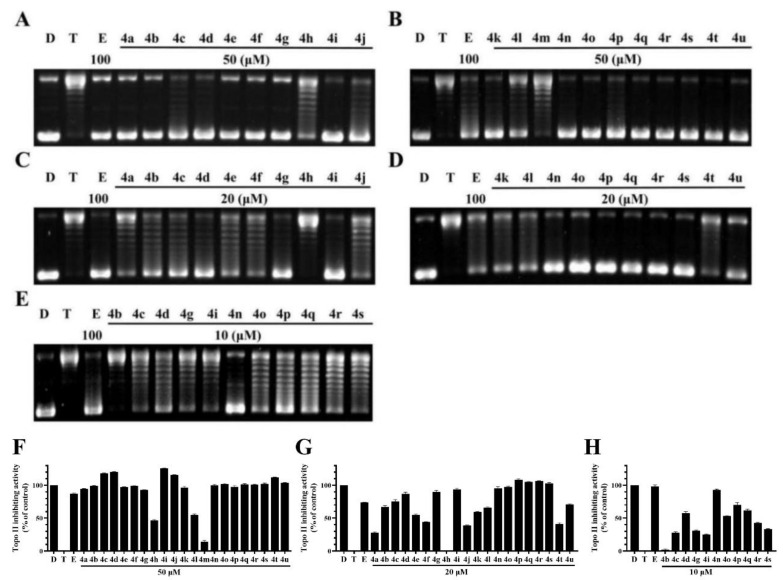
Topo II inhibiting activity of BDHs in Topo II-mediated DNA relaxation assay. lane D: pBR322 DNA; lane T: pBR322 DNA + Topo II; lane E: pBR322 DNA + Topo II + etoposide (100 μM); other lanes: pBR322 DNA + Topo II + compounds. (**A**,**B**) BCHs at concentration of 50 μM; (**C**,**D**) BCHs at concentration of 20 μM; (**E**) BCHs at concentration of 10 μM; (**F**–**H**) semi-quantification of Topo II inhibiting activity of BCHs at 50, 20, 10 μM, respectively, with positive control (lane D: pBR322 DNA) as 100%, negative control (lane T: pBR322 DNA + Topo II) as 0% and comparative control (lane E: pBR322 DNA + Topo II + etoposide 100 μM).

The Topo II-mediated DNA relaxation assay was performed to evaluate the inhibitory effect of BCHs against the catalytic activity of Topo II. Etoposide was used as the comparative control and pBR322 DNA plasmid as the substrate [30]. BCHs were supposed to inhibit DNA relaxation catalyzed by Topo II. The decrease of pBR322 and the increase of relaxed DNA were indicators of catalytic activity of Topo II. According to the western blot and semi-quantification results (Figure 3), it was found that most BCHs showed strong Topo II inhibition activity at 50 μL (Figure 3), while **4d**, **4n**, **4o**, **4p**, and **4q** showed relatively stronger inhibitory potency at 10 μL (Figure 3) than the others. Generally, compounds with an electron-donating group such as (-C(CH_3_)_2_, -CH_2_CH_3_, -CH_3_, -OCH_3_) at R_2_ position showed stronger inhibitory activity on Topo II than that of compounds with an electron-withdrawing group (-NO_2_, -CN). Substitutions at R_2_ with electron-withdrawing groups, such as-NO_2_ (**4k**) and -CN (**4u**) groups, significantly impaired the inhibitory potency, while a bromine (compounds **4c**, **4d**) and chlorine (compounds **4i**, **4j**) atom at the position were basically tolerated. The impact of diversification at R_1_ position to the inhibitory activity were found to be inconclusive, as a methyl group or a fluorine atom were moderately tolerated. Based on **4g**, more molecules with electron-donating groups at R_2_ were prepared and screened. Among all compounds of the series, **4n** was identified as the most potent one, which inhibited 94.9% at 20 μM and 93.1% at 10 μM of the Topo II’s catalytic activity in DNA relaxation, compared with the positive control group without Topo II.

### 2.3. Anti-Proliferative Activity of BCHs on Tumor Cells

Given that etoposide, a typical Topo II inhibitor, can be served as an effective anti-cancer drug, we moved on to evaluate the anti-tumor activity of our compounds on tumor cells. The cytotoxicity of BCHs was evaluated using the MTT assay against four human tumor cell lines, including HepG2 (human hepatoma cell line), A549 (human lung cancer cell line), LNCaP (human prostate cancer cell line), MG63 (human osteosarcoma cell line). Etoposide was included as the comparative control. The inhibitory activities are expressed as IC_50_ and listed in Table 1.

Most of the tested compounds displayed strong antiproliferative activity in the four tested cancer cell lines. In particular, for BCHs with the best inhibitory activity against A549 cells, the IC_50_ values of **4a** and **4h** reached 2.75 μM and 2.86 μM, and the IC_50_ values of **4d** and **4n** with better inhibitory activity against Topo II also reached 3.55 μM and 3.81 μM, respectively. Notably, **4a** showed approximately 4-fold increase of inhibition potency, compared with that of etoposide on A549 cells and LNCaP cells. Cellular permeability and metabolic stability of compounds in different tumor cell lines could contribute to the gap between molecular and cellular pharmacological activity. The anti-proliferative effect of the synthesized compounds was largely consistent with their Topo II catalytic inhibition activity, except for compound **4h**, which was possibly due to an off-target mechanism. The anti-proliferative activity of these series of compounds was generally better than the Topo II inhibitors we previously reported [31]. Compared with the benzimidazole or chalcone pharmacophore separately, which represented by **2a** and chalcone, respectively, this series of hybrids showed remarked improvement in anti-proliferative activity against tumor cell lines. This work was good practice of the classic pharmacophore hybridization strategy, which was also employed in another series of Topo II poisons containing both the pharmacophores of merbarone and etoposide, with similar anti-proliferative activity in cancer cell lines [28]. After balanced consideration of both inhibitory activities against Topo II catalytic activity and cell proliferation, **4d** and **4n** were chosen for further studies.

### 2.4. BCHs as Non-Intercalative Topo II Catalytic Inhibitors

**Figure 4:**
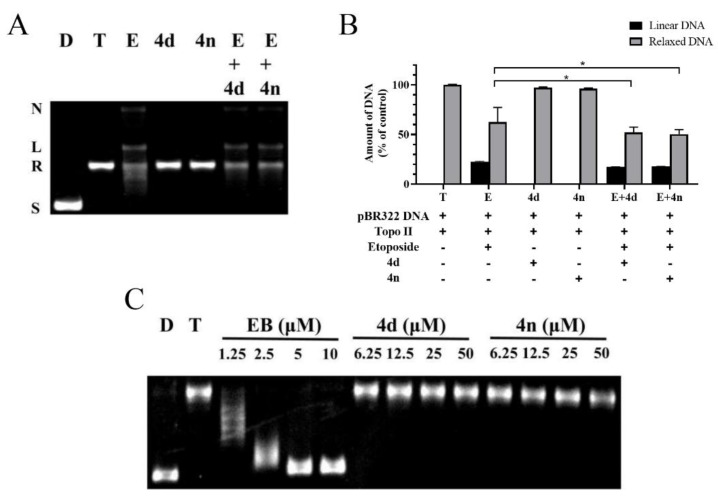
(**A**) Western blot gel of the Topo II-DNA cleavage assay. Lane 1-2: control group of supercoiled pBR322 DNA without or with Topo II; Lane 3-5: addition of etoposide (100 μM), 4d or 4n (50 μM) on Topo II with supercoiled pBR322 DNA; Lane 6-7: pretreatment of pBR322 DNA and Topo II with 4d or 4n (50 μM) followed by addition of etoposide. In each lane, the positions of supercoiled DNA (S), relaxed DNA (R), linear DNA (L) and nicked DNA (N) are indicated. (**B**) Semi-quantification of the western blot gel in panel A. Data were presented as Mean ± SD (n = 3). * *p* < 0.05 indicates statistically significant difference of relaxed DNA compared with group E (Student’s *t*-test). (**C**) The unwinding assay of compounds. Lane D: pBR322 DNA; lane T: pBR322 DNA + Topo I; other lanes: pBR322 DNA + Topo I + 4d, 4n or EB of different concentrations.

Since two typical mechanisms-of-action of Topo II inhibitors exist, assays for a mechanistic study were performed. One mode of mechanism-of-action of Topo II poisons is to stabilize the Topo II-DNA complex and promote the formation of linear DNA, while the other mode is to interfere the binding of ATP to the enzyme and block the catalytic activity of the ATPase. The Topo II-mediated DNA cleavage assay was employed to determine the mode of action, with etoposide, a typical Topo II poison, as the comparative control. In the assay, proteinase K was added to cleavage Topo II-DNA complex stabilized by the Topo II poison etoposide, which eventually produced linear DNA. According to the results, the stains in the gel which represented linear DNA was observed in the etoposide group. In contrast, no obvious linear form of DNA was noticed in neither **4d** nor **4n** group (Figure 4A,B), indicating that **4d** and **4n** were not a Topo II poison at concentration of 50 μM. More importantly, pretreatment of **4d** or **4n** (group E + 4d, E + 4n) significantly decreased the amount of linear DNA compared with group E. It implied that etoposide could be antagonized by **4d** or **4n,** suggesting that **4d**, **4n** and the other BCHs of the series were Topo II catalytic inhibitors.

In order to verify whether our Topo II catalytic inhibitors function as DNA intercalators or non-intercalators, a Topo I-mediated DNA unwinding assay was carried out with **4d** and **4n**. In this assay, the supercoiled DNA could be completely relaxed by excessive Topo I (Figure 4C). However, addition of DNA intercalators, such as ethidium bromide (EB), could block the relaxation. As shown in Figure 4C, the relaxation of DNA was partially inhibited by different concentrations of EB, while no inhibiting effect was observed in groups adding **4d** or **4n**, even at as high as 50 μM.

In view of these results above, this series of BCHs, exemplified by **4d** and **4n**, inhibited Topo II enzyme activity and suppress tumor cell proliferation. But it did not stabilize Topo II-DNA complex as Topo II poisons nor intercalate with DNA. Thus, it could be agreeably inferred that this series of BCHs acted as non-intercalative Topo II catalytic inhibitors.

### 2.5. Inhibition of Clonogenic Survival of A549 Cells by ***4d*** and ***4n***

**Figure 5:**
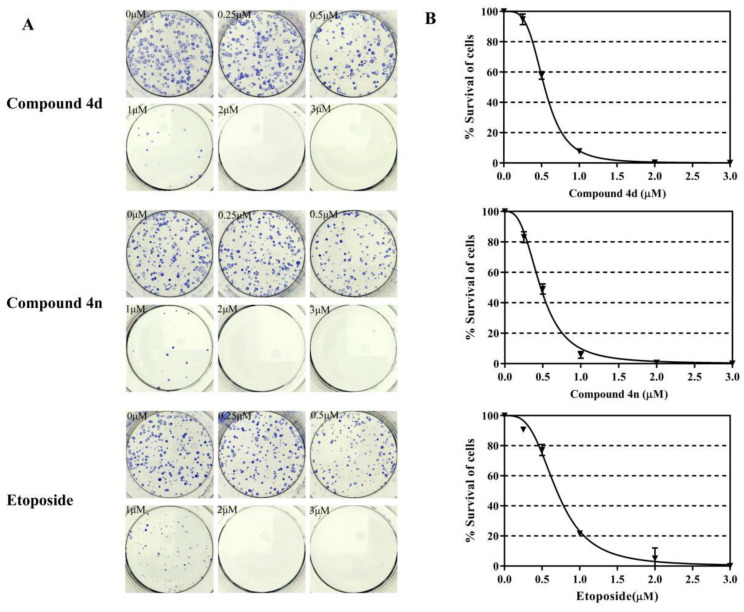
Compound **4d** and **4n** inhibited the clonogenic survival of A549 cells. (**A**) The developed colonies were counted and compared with the untreated control as survival percentage. (**B**) Dose-dependent curves of **4n**, **4n** and etoposide. Data represented here are the mean of three independent experiments performed in triplicates.

The clonogenic cell viability test is a long-term cell viability assay, which determines whether the cells can proliferate infinitely, thus maintaining their reproductive capacity to form colonies. This assay was used to examine the anti-colony forming ability of **4d** and **4n** in A549 cells. A549 cells were incubated for 24 h with different concentrations of **4d** and **4n** (0–3 μM), and the assay was performed according to previously described protocols [32,33]. The survival of A549 cells decreased to 10% after 1 μM treatment of **4d** or **4n**, even the survival of A549 cells was almost 0% in the 2 μM treatments of **4d** or **4n** (Figure 5). Etoposide also showed good anti-colony forming ability in A549 cells. The IC_50_ values for inhibition of A549 cells clonogenic by compound **4d** and **4n** were 0.54 μM and 0.47 μM respectively, while etoposide was 0.70 μM. According to these results, we concluded that **4d** and **4n** had strong inhibition of A549 cell colony formation after a short exposure.

### 2.6. Inhibition of Wound Healing in A549 Cell by ***4d*** and ***4n***

**Figure 6:**
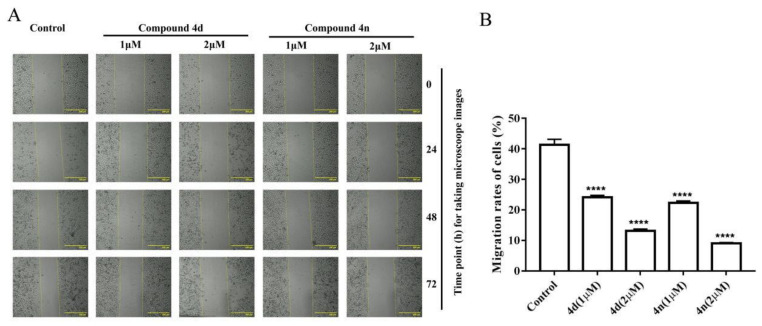
Compound **4d** and **4n** inhibited wound healing of A549 cells. (**A**) The wound was captured by JuLI Intelligent fluorescent cell monitor at different time points (0, 24, 48 and 72 h). Scale bar: 500 μm. (**B**) Quantification of the cell scrape results using ImageJ software. Values are means ± SD, **** indicates *p* < 0.0001 (Student’s *t*-test).

The wound healing assay is a classic and common method used to investigate the effect of antitumor drugs on tumor migration. According to previous studies, scratch-wound healing assay was used to assess the effect of compounds on the migration and interaction capacity of A549 cells. A sterile tip scratched the monolayer of cells and a wound was created. Then it was inspected after the treatments of 1 μM and 2 μM **4d** or **4n**. The untreated control cells migrated across the well with time. Obviously, the migration ability of treated cells was time dependent. The scratch gaps in **4d**- and **4n**-treated A549 cells were wider than control with increased treating time (Figure 6). Combining the results of cytotoxicity, clonogenic survival and scratch-wound assay, we concluded that compound **4d** and **4n** were able to inhibit tumor viability, colony formation, and migration of A549 cells.

### 2.7. Cell Apoptosis of A549 Cells by ***4d*** and ***4n***

**Figure 7:**
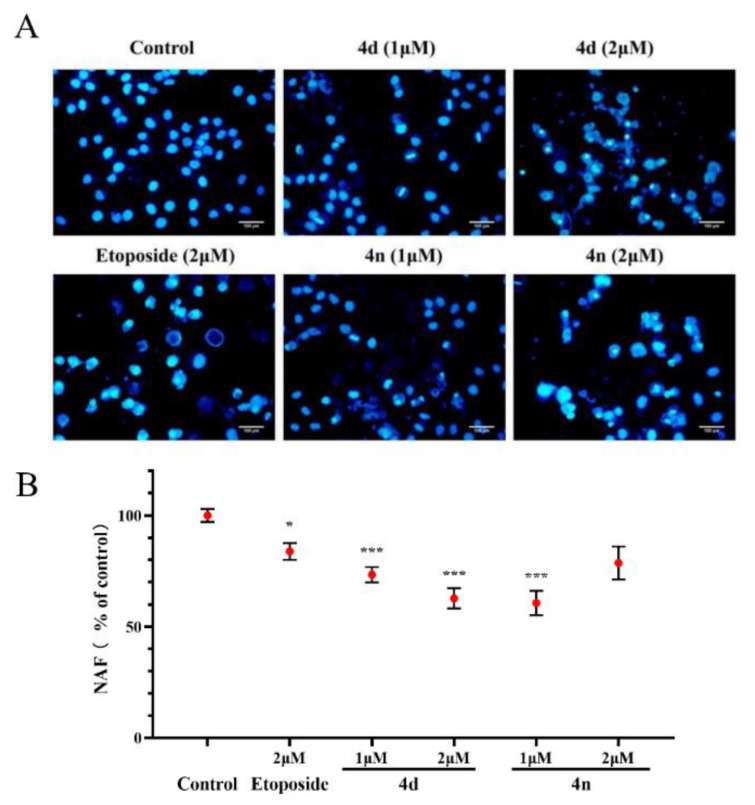
Morphological observation on A549 cells by DAPI staining. (**A**) DAPI staining of A549 cells treated with **4d** and **4n** (1 μM, 2 μM), with etoposide (2 μM) as a comparative control. (**B**) Morphological alterations were semi-quantification by NAF calculated by the product of nuclear area and circularity. Values (% of control) were presented as means ± SD (n = 3), labeled with * *p* < 0.05 or with *** *p* < 0.001 compared with the control group (Student’s *t*-test). Scale bar: 100 µm.

We also investigated the effects of compounds in inducing A549 cells apoptosis using fluorescence microscope by staining with DAPI [34,35]. The results in Figure 7A indicate that the nuclear structure of the control cells was intact with round shape while the A549 cells treated with the **4d** and **4n** showed nuclear concentration and micronuclei formation. Morphological alterations were semi-quantified with Nuclear area factor (NAF), which was regarded as an early indicator of cell morphological changes occurring during apoptosis (Figure 7B). It was calculated by the product of average nuclear area and circularity. According to the figure, the BCHs could probably induce apoptosis of A549 cells compared with the control group.

### 2.8. Molecular Docking

**Figure 8:**
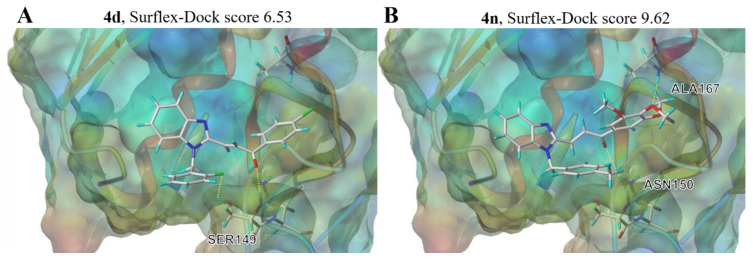
Schematic illustration of proposed binding mode of **4d** and **4n** with the ATP binding pocket of Topo II (PDB code: 1ZXM) (**A**), compound **4d**; (**B**), compound **4n**.

To further rationalize the inhibitory activity and SAR of BCHs, a flexible ligand receptor docking investigation of **4d** and **4n** was carried out on SYBYL platform with a Surflex-Dock module. The crystal structure data of Topo II protein was originated from the protein data bank (PDB code: 1ZXM). Various binding pockets had been searched for and finally the ATP-binding pocket was identified as the optimal binding site represented by high total scores. **4d** formed two hydrogen bounds with residues SER149 via the fluorine atom and the carbonyl group, while **4n** interacted with the enzyme via two hydrogen bounds with ASN150 and ALA167 through the carbonyl group and methoxy group of the chalcone motif. Both compounds were evaluated with higher total score (6.53 and 9.62 of **4d** and **4n**, respectively) than that of etoposide (total score 6.52) according to the docking results (Supplementary Materials Table S1). As we can see in Figure 8, both hybrid **4d** and **4n** were found to locate in the cavity which were supposed to accommodate ATP, which was demonstrated in the cocrystal structure of adenosine diphosphate (ADP) and the N-terminal ATPase domain of Topo II (Supplementary Materials Figures S1–S3). Given that, it can be inferred that inhibition mode of hybrid **4d** and **4n** against Topo II was catalytic inhibition through occupying ATP binding pocket of N-terminal ATPase domain of Topo II, and modification of may furtherly promote the inhibitory activity.

## 3. Materials and Methods

### 3.1. Chemistry

#### 3.1.1. Synthesis and Characterization

^1^H- and ^13^C-NMR spectra were recorded using TMS as the internal standard in CDCl_3_ with a Bruker BioSpin GmbH (Bruker, Billerica, MA, USA) spectrometer at 400 MHz for ^1^H and 101 MHz for ^13^C. Mass spectra (ESI-MS) were recorded on a 4000 QTRAP type LC/MS (SCIEX, Framingham, MA, USA). The purities of final compounds were higher than 95%, confirmed by an analytical HPLC on a dual pump LC-20AB system (Shimadzu, Tokyo, Japan). HPLC conditions: methanol/H_2_O = 80/20 (*v*/*v*); flow rate, 0.5 mL/min; column, Ultimate XB-C18.

#### 3.1.2. Procedure for the Synthesis of Intermediate **1a**

A solution of *o*-phenylenediamine (10.8 g, 10 mmol, 1 equiv) and glycolic acid (22.8 g, 30 mmol, 3 equiv) in 4N HCl (70 mL) was stirred at 100 °C for 6 h. After the reaction was complete, the reaction mixture was cooled to room temperature, and an appropriate amount of NaHCO_3_ was added to neutralize hydrochloric acid, and an off-white solid precipitate was formed. The precipitated solid was filtered, washed with water and dried in a vacuum oven to get the crude product, which then was purified by column chromatography (200 mesh silica gel, ethyl acetate/ petroleum ether = 8:1) to yield the title compound **1a**.

#### 3.1.3. General Procedure for the Synthesis of Intermediates **2a** and **2b**

To a solution of **1a** (2.96 g, 20 mmol, 1 equiv) in DMF (30 mL) was added the appropriate benzyl bromide (40 mmol, 2 equiv) and K_2_CO_3_ (100 mmol, 5 equiv), and the reaction mixture was stirred at room temperature overnight. After removal of the solvent under vacuum the residue was partitioned between DCM (70 mL) and H_2_O (70 mL). The organic layer was separated, and the aqueous layer was extracted with DCM (70 mL × 2). The combined organic layers were concentrated in vacuo to provide a crude product, which was purified by column chromatography (200 mesh silica gel, ethyl acetate/petroleum ether = 8:1) to yield the title compounds **2a** and **2b**.

*(1-(2-Fluorobenzyl)-1H-benzo[d]imidazol-2-yl)methanol* (**2a**). Light yellow thick liquid, yield: 47%. ^1^H- NMR (CDCl_3_) δ 7.57–7.52 (m, 1H), 7.43–7.36 (m, 1H), 7.34–7.26 (m, 1H), 7.26–7.17 (m, 3H), 7.14–7.07 (m, 2H), 5.57 (d, *J* = 1.1 Hz, 2H), 4.70 (d, *J* = 5.5 Hz, 2H), 3.92 (t, *J* = 5.5 Hz, 1H). ^13^C-NMR (CDCl_3_) δ 160.6 (d, *J* = 203.5 Hz), 155.3, 141.1, 136.4, 130.1 (d, *J* = 72.7 Hz), 129.4 (d, *J* = 6.5 Hz), 124.7 (d, *J* = 16.2 Hz), 124.2, 123.1, 119.4, 115.6 (d, *J* = 16.2 Hz), 111.2, 57.9, 45.2(d, *J* = 4.0 Hz). MS (ESI): calcd for C_15_H_13_FN_2_O [M + H]^+^ 257.1, found 257.1. HPLC purity: 97.5%.

*(1-(4-Methylbenzyl)-1H-benzo[d]imidazol-2-yl)methanol* (**2b**). Light yellow thick liquid, yield: 88%. ^1^H- NMR (CDCl_3_) δ 7.57–7.52 (m, 1H), 7.43–7.37 (m, 1H), 7.22 (pd, *J* = 7.1, 1.5 Hz, 2H), 7.09–7.00 (m, 4H), 5.54 (t, *J* = 0.9 Hz, 2H), 4.70 (d, *J* = 5.5 Hz, 2H), 3.92 (t, *J* = 5.5 Hz, 1H). ^13^C-NMR (CDCl_3_) δ 155.2, 141.1, 136.8, 136.5, 134.0, 129.2, 127.3, 124.2, 123.1, 119.4, 111.2, 57.9, 47.4, 21.0. MS (ESI): calcd for C_16_H_16_N_2_O [M + H]^+^ 253.1, found 253.1. HPLC purity: 95.9%.

#### 3.1.4. General Procedure for the Synthesis of Intermediates **3a** and **3b**

A solution of **2a** or **2b** (10 mmol, 1 equiv) in DCM (20 mL) was stirred at 0 °C for 15 min, then added Dess-Martin periodinane (11 mmol, 1.1 equiv) was added and the mixture stirred at 0 °C for another 1 h. After the reaction was complete, it was returned to room temperature and an appropriate amount of a saturated Na_2_S_2_O_3_ solution was added to remove the excess Dess-Martin periodinane. Next the mixture was diluted with DCM (70 mL) and H_2_O (70 mL). The organic layer was separated, and the aqueous layer was extracted with DCM (70 mL × 2). The combined organic layers were concentrated in vacuo to provide a crude product, which was purified by column chromatography (200 mesh silica gel, ethyl acetate/petroleum ether = 10:1) to yield the title compounds **3a** and **3b**.

*1-(2-Fluorobenzyl)-1H-benzo[d]imidazole-2-carbaldehyde* (**3b**). Light yellow semisolid, yield: 78%. ^1^H- NMR (CDCl_3_) δ 9.78 (1H, s), 7.69 (dd, *J* = 6.5, 1.4 Hz, 1H), 7.44 (dd, *J* = 7.2, 1.4 Hz, 1H), 7.34–7.20 (m, 4H), 7.14–7.07 (m, 2H), 5.59 (d, *J* = 1.1 Hz, 2H). ^13^C-NMR (CDCl_3_) δ 182.0, 160.6 (d, *J* = 203.4 Hz), 151.1, 139.2, 137.4, 130.1 (d, *J* = 65.7 Hz), 129.4 (d, *J* = 6.6 Hz), 124.7 (d, *J* = 5.7 Hz), 124.2 (d, *J* = 16.2 Hz), 123.3, 120.1, 115.6 (d, *J* = 16.2 Hz), 113.1, 44.5 (d, *J* = 4.0 Hz). MS (ESI): calcd for C_15_H_11_FN_2_O [M + H]^+^ 255.1, found 255.1. HPLC purity: 96.5%.

*1-(4-Methylbenzyl)-1H-benzo[d]imidazole-2-carbaldehyde* (**4b**). Light yellow semisolid, yield: 56%. ^1^H-NMR (CDCl_3_) δ 9.61 (s, 1H), 7.69 (dd, *J* = 6.5, 1.4 Hz, 1H), 7.44 (dd, *J* = 7.2, 1.4 Hz, 1H), 7.32–7.20 (m, 2H), 7.09–7.00 (m, 4H), 5.54 (t, *J* = 1.0 Hz, 2H), 2.34 (s, 2H). ^13^C-NMR (CDCl_3_) δ 182.2, 150.9, 139.2, 137.6, 136.8, 133.7, 129.2, 127.3, 124.6, 123.3, 120.1, 113.1, 46.5, 21.0. MS (ESI): calcd for C_16_H_14_N_2_O [M + H]^+^ 251.1, found 251.1. HPLC purity: 95.7%.

#### 3.1.5. General Procedure for the Synthesis of Compounds **4a**–**4u**

Compounds **3a** or **3b** (2 mmol, 1 equiv) and an appropriate acetophenone (2 mmol, 1 equiv) were dissolved in EtOH (10 mL) and 10% NaOH (0.5 mL) was added slowly. Then, the mixture was stirred at room temperature for 4 h. The solid was filtered off and recrystallized from ethanol to give a light-yellow solid.

*(E)-3-(1-(2-Fluorobenzyl)-1H-benzo[d]imidazol-2-yl)-1-(p-tolyl)prop-2-en-1-one* (**4a**). 4′-Methyl-acetophenone and **3a** were used as reactants to give **4a**. Yield: 73%, mp: 167.3–168.5 °C. ^1^H-NMR (CDCl_3_) δ 8.40 (d, *J* = 15.0 Hz, 1 H), 8.04 (d, *J* = 8.2 Hz, 2 H), 7.90–7.85 (m, 2 H), 7.39–7.27 (m, 6 H), 7.15–7.10 (m, 1 H), 7.01 (t, *J* = 7.1 Hz, 1 H), 6.80 (t, *J* = 7.0 Hz, 1 H), 5.60 (s, 2 H), 2.44 (s, 3 H). ^13^C-NMR (CDCl_3_) δ 188.5, 160.1 (d, *J* = 247.0 Hz), 148.8, 144.5, 143.3, 135.8, 134.9, 130.0 (d), 129.5, 129.0, 128.7, 128.0 (d), 127.4, 124.8 (d, *J* = 3.6 Hz), 124.4, 123.7, 122.8 (d, *J* = 14.3 Hz), 120.4, 115.9 (d, *J* = 21.0 Hz), 110.2, 41.1 (d, *J* = 5.2 Hz), 21.7. HRMS (ESI): calcd for C_24_H_19_FN_2_O [M + H]^+^ 371.1554, found 371.1571. HPLC purity: 95.8%.

*(E)-3-(1-(4-Methylbenzyl)-1H-benzo[d]imidazol-2-yl)-1-(p-tolyl)prop-2-en-1-one* (**4b**). 4′-Methyl-acetophenone and **3b** were used as reactants to give **4b**. Yield: 82%, mp: 207.6–209.2 °C. ^1^H-NMR (CDCl_3_) δ 8.37 (d, *J* = 15.0 Hz, 1 H), 8.03 (d, *J* = 8.2 Hz, 2 H), 7.88–7.83 (m, 2 H), 7.37–7.28 (m, 5 H), 7.10 (d, *J* = 8.0 Hz, 2 H), 7.01 (d, *J* = 8.0 Hz, 2 H), 5.51 (s, 2 H), 2.44 (s, 3 H), 2.30 (s, 3 H). ^13^C-NMR (CDCl_3_) δ 188.6, 148.7, 144.5, 143.3, 138.0, 136.0, 134.9, 132.7, 129.8, 129.5, 129.0, 128.5, 127.7, 126.3, 124.2, 123.6, 120.3, 110.3, 47.0, 21.7, 21.1. HRMS (ESI): calcd for C_25_H_22_N_2_O [M + H]^+^ 367.1805, found 367.1809. HPLC purity: 96.2%.

*(E)-1-(4-Bromophenyl)-3-(1-(4-methylbenzyl)-1H-benzo[d]imidazol-2-yl)prop-2-en-1-one* (**4c**). 4′-Bromo-acetophenone and **3b** were used as reactants to give **4c**. Yield: 81%, mp: 194.5–196.3 °C. ^1^H-NMR (CDCl_3_) δ 8.31 (d, *J* = 15.0 Hz, 1 H), 7.97 (d, *J* = 8.5 Hz, 2 H), 7.89–7.84 (m, 2 H), 7.65 (d, *J* = 8.5 Hz, 2 H), 7.37–7.30 (m, 3 H), 7.11 (d, *J* = 8.1 Hz, 2 H), 7.00 (d, *J* = 8.0 Hz, 2 H), 5.51 (s, 2 H), 2.30 (s, 3 H). ^13^C-NMR (CDCl_3_) δ 188.0, 148.4, 143.3, 138.1, 136.1, 136.1, 132.6, 132.1, 130.3, 129.8, 128.6, 127.6, 126.3, 124.5, 123.7, 120.4, 110.4, 47.0, 21.1. HRMS (ESI): calcd for C_24_H_19_BrN_2_O [M + H]^+^ 431.0754, found 431.0751. HPLC purity: 95.9%.

*(E)-1-(4-Bromophenyl)-3-(1-(2-fluorobenzyl)-1H-benzo[d]imidazol-2-yl) prop -2-en-1-one* (**4d**). 4′-Bromo-acetophenone and **3a** were used as reactants to give **4d**. Yield: 78%, mp: 156.7–157.5 °C. ^1^H-NMR (CDCl_3_) δ 8.34 (d, *J* = 14.9 Hz, 1 H), 7.99 (d, *J* = 8.6 Hz, 2 H), 7.92–7.84 (m, 2 H), 7.66 (d, *J* = 8.5 Hz, 2 H), 7.40–7.28 (m, 4 H), 7.16–7.10 (m, 1 H), 7.02 (t, *J* = 7.5 Hz, 1 H), 6.80 (t, *J* = 7.6 Hz, 1 H), 5.60 (s, 2 H). ^13^C-NMR (CDCl_3_) δ 187.9, 160.1 (d, *J* = 246.9 Hz), 148.4, 143.3, 136.1, 135.9, 132.1, 130.3, 130.1 (d), 128.8, 128.3, 128.1 (d), 127.9, 124.8 (d), 124.6, 123.9, 122.7 (d), 120.5, 115.8 (d), 110.2, 41.1 (d). HRMS (ESI): calcd for C_23_H_16_BrFN_2_O [M + H]^+^ 435.0503, found 435.0508. HPLC purity: 98.4%.

*(E)-1-(4-Fluorophenyl)-3-(1-(4-methylbenzyl)-1H-benzo[d]imidazol-2-yl)prop-2-en-1-one* (**4e**). 4′-Fluoro-acetophenone and **3b** were used as reactants to give **4e**. Yield: 79%, mp: 206.7–207.4 °C. ^1^H-NMR (CDCl_3_) δ 8.34 (d, *J* = 15.0 Hz, 1 H), 8.15 (dd, *J* = 8.8, 5.4 Hz, 2 H), 7.88–7.83 (m, 2 H), 7.38–7.29 (m, 3 H), 7.18 (t, *J* = 8.6 Hz, 2 H), 7.11 (d, *J* = 7.9 Hz, 2 H), 7.01 (d, *J* = 8.0 Hz, 2 H), 5.51 (s, 2 H), 2.30 (s, 3 H). ^13^C-NMR (CDCl_3_) δ 187.5, 166.0 (d), 148.5, 143.3, 138.0, 136.0, 133.8 (d), 132.6, 131.5 (d), 129.8, 128.3, 127.9, 126.3, 124.4, 123.7, 120.4, 115.9 (d), 110.4, 47.0, 21.1. HRMS (ESI): calcd for C_24_H_19_FN_2_O [M + H]^+^ 371.1554, found 371.1558. HPLC purity: 98.6%.

*(E)-3-(1-(2-Fluorobenzyl)-1H-benzo[d]imidazol-2-yl)-1-(4-fluorophenyl)prop-2-en-1-one* (**4f**). 4′-Fluoro-acetophenone and **3a** were used as reactants to give **4f**. Yield: 70%, mp: 165.9–167.2 °C. ^1^H-NMR (CDCl_3_) δ 8.37 (d, *J* = 14.9 Hz, 1 H), 8.19–8.14 (m, 2 H), 7.88 (t, *J* = 11.7 Hz, 2 H), 7.39–7.27 (m, 4 H), 7.16 (dt, *J* = 18.5, 8.6 Hz, 3 H), 7.02 (t, *J* = 7.5 Hz, 1 H), 6.80 (t, *J* = 7.2 Hz, 1 H), 5.60 (s, 2 H). ^13^C-NMR (CDCl_3_) δ 187.4, 166.0 (d), 160.1 (d), 148.5, 143.3, 135.8, 133.8 (d), 131.5 (d), 130.1 (d), 128.1, 128.0 (d), 128.0, 124.8 (d), 124.5, 123.8, 122.8 (d), 120.4, 116.0 (d), 115.8 (t), 110.2, 41.1 (d). HRMS (ESI): calcd for C_23_H_16_F_2_N_2_O [M + H]^+^ 375.1303, found 375.1302. HPLC purity: 99.3%.

*(E)-1-(4-Methoxyphenyl)-3-(1-(4-methylbenzyl)-1H-benzo[d]imidazol-2-yl)prop-2-en-1-one* (**4g**). 4′-Methoxyacetophenone and **3b** were used as reactants to give **4g**. Yield: 80%, mp: 196.2–197.6 °C. ^1^H-NMR (CDCl_3_) δ 8.37 (d, *J* = 15.0 Hz, 1 H), 8.13 (d, *J* = 8.9 Hz, 2 H), 7.87–7.82 (m, 2 H), 7.37–7.27 (m, 3 H), 7.10 (d, *J* = 7.9 Hz, 2 H), 7.03–6.97 (m, 4 H), 5.51 (s, 2 H), 3.90 (s, 3 H), 2.29 (s, 3 H). ^13^C-NMR (CDCl_3_) δ 187.3, 164.0, 148.8, 143.3, 138.0, 136.0, 132.7, 131.2, 130.5, 129.8, 128.5, 127.4, 126.3, 124.2, 123.5, 120.3, 114.0, 110.3, 55.6, 47.0, 21.1. HRMS (ESI): calcd for C_25_H_22_N_2_O_2_ [M + H]^+^ 383.1754, found 383.1760. HPLC purity: 96.7%.

*(E)-3-(1-(2-Fluorobenzyl)-1H-benzo[d]imidazol-2-yl)-1-(4-methoxyphenyl)prop-2-en-1-one* (**4h**). 4′-Methoxyacetophenone and **3a** were used as reactants to give **4h**. Yield: 73%, mp: 155.8–157.3 °C. ^1^H-NMR (CDCl_3_) δ 8.40 (d, *J* = 14.9 Hz, 1 H), 8.14 (d, *J* = 8.8 Hz, 2 H), 7.89–7.84 (m, 2 H), 7.34 (dt, *J* = 26.0, 8.6 Hz, 4 H), 7.15–7.10 (m, 1 H), 7.00 (dd, *J* = 11.3, 8.3 Hz, 3 H), 6.80 (t, *J* = 7.6 Hz, 1 H), 5.60 (s, 2 H), 3.90 (s, 3 H). ^13^C-NMR (CDCl_3_) δ 187.3, 164.0, 160.1 (d, *J* = 247.0 Hz), 148.9, 143.3, 135.8, 131.3, 130.4, 130.0 (d), 128.7, 128.0 (d), 127.0, 124.8 (d), 124.3, 123.7, 122.8 (d), 120.3, 115.8 (d), 114.0, 110.2, 55.6, 41.1 (d). HRMS (ESI): calcd for C_24_H_19_FN_2_O_2_ [M + H]^+^ 387.1503, found 387.1504. HPLC purity: 99.8%.

*(E)-1-(4-Chlorophenyl)-3-(1-(4-methylbenzyl)-1H-benzo[d]imidazol-2-yl)prop-2-en-1-one* (**4i**). 4′-Chloro-acetophenone and **3b** were used as reactants to give **4i**. Yield: 84%, mp: 206.4–208.1 °C. ^1^H-NMR (CDCl_3_) δ 8.32 (d, *J* = 15.0 Hz, 1 H), 8.05 (d, *J* = 8.5 Hz, 2 H), 7.89–7.83 (m, 2 H), 7.48 (d, *J* = 8.5 Hz, 2 H), 7.38–7.29 (m, 3 H), 7.11 (d, *J* = 7.9 Hz, 2 H), 7.00 (d, *J* = 8.0 Hz, 2 H), 5.51 (s, 2 H), 2.30 (s, 3 H). ^13^C-NMR (CDCl_3_) δ 187.8, 148.4, 143.3, 140.0, 138.1, 136.1, 135.7, 132.6, 130.2, 129.8, 129.1, 128.5, 127.7, 126.3, 124.5, 123.7, 120.4, 110.4, 47.0, 21.1. HRMS (ESI): calcd for C_24_H_19_ClN_2_O [M + H]^+^ 387.1259, found 387.1273. HPLC purity: 98.2%.

*(E)-1-(4-Chlorophenyl)-3-(1-(2-fluorobenzyl)-1H-benzo[d]imidazol-2-yl)prop-2-en-1-one* (**4j**). 4′-Chloro-acetophenone and **3a** were used as reactants to give **4j**. Yield: 78%, mp: 166.2–167.3 °C. ^1^H-NMR (CDCl_3_) δ 8.35 (d, *J* = 14.9 Hz, 1 H), 8.07 (d, *J* = 8.5 Hz, 2 H), 7.92–7.84 (m, 2 H), 7.49 (d, *J* = 8.5 Hz, 2 H), 7.33 (ddd, *J* = 19.5, 12.8, 7.8 Hz, 4 H), 7.16–7.10 (m, 1 H), 7.01 (t, *J* = 7.5 Hz, 1 H), 6.80 (t, *J* = 7.7 Hz, 1 H), 5.60 (s, 2 H). ^13^C-NMR (CDCl_3_) δ 187.7, 160.1 (d), 148.5, 143.3, 140.0, 135.9, 135.7, 130.2, 130.1 (d), 129.1, 128.2, 128.1 (d), 127.9, 124.8 (d), 124.6, 123.9, 122.8 (d), 120.5, 115.8 (d), 110.2, 41.1 (d). HRMS (ESI): calcd for C_23_H_16_ClFN_2_O [M + H]^+^ 391.1008, found 391.1004. HPLC purity: 97.9%.

*(E)-3-(1-(4-Methylbenzyl)-1H-benzo[d]imidazol-2-yl)-1-(4-nitrophenyl)prop-2-en-1-one* (**4k**). 4′-Nitro-acetophenone and **3b** were used as reactants to give **4k**. Yield: 76%, mp: 209.8–211.5 °C. ^1^H-NMR (CDCl_3_) δ 8.33 (dd, *J* = 15.0, 8.8 Hz, 3 H), 8.24 (d, *J* = 8.8 Hz, 2 H), 7.92–7.85 (m, 2 H), 7.39–7.32 (m, 3 H), 7.12 (d, *J* = 8.0 Hz, 2 H), 7.00 (d, *J* = 8.1 Hz, 2 H), 5.52 (s, 2 H), 2.31 (s, 3 H). ^13^C-NMR (CDCl_3_) δ 187.6, 150.4, 148.0, 143.3, 142.0, 138.2, 136.1, 132.6, 129.8, 129.7, 129.7, 127.0, 126.2, 124.8, 123.9, 123.9, 120.5, 110.4, 47.0, 21.1. HRMS (ESI): calcd for C_24_H_19_N_3_O_3_ [M + H]^+^ 398.1499, found 398.1492. HPLC purity: 98.0%.

*(E)-1-(3,4-Dimethoxyphenyl)-3-(1-(4-methylbenzyl)-1H-benzo[d]imidazol-2-yl)prop-2-en-1-one* (**4l**). 3′,4′-Dimethoxyacetophenone and **3b** were used as reactants to give **4l**. Yield: 82%, mp: 193.8–195.4 °C. ^1^H-NMR (CDCl_3_) δ 8.39 (d, *J* = 14.9 Hz, 1 H), 7.88–7.82 (m, 2 H), 7.66 (s, 1 H), 7.33 (dd, *J* = 14.4, 6.5 Hz, 3 H), 7.15–6.98 (m, 5 H), 6.91 (dd, *J* = 16.3, 8.3 Hz, 1 H), 5.52 (s, 2 H), 3.97 (s, 6 H), 2.30 (s, 3 H). ^13^C-NMR (CDCl_3_) δ 187.2, 153.8, 149.3, 148.8, 143.3, 138.0, 136.0, 132.7, 130.6, 129.8, 128.2, 127.3, 126.3, 124.2, 123.9, 123.6, 120.2, 110.7, 110.3, 110.1, 56.1, 47.0, 21.1. HRMS (ESI): calcd for C_26_H_24_N_2_O_3_ [M + H]^+^ 413.1867, found 413.1866. HPLC purity: 95.0%.

*(E)-1-(3,4-Dimethoxyphenyl)-3-(1-(2-fluorobenzyl)-1H-benzo[d]imidazol-2-yl)prop-2-en-1-one* (**4m**). 3′,4′-Dimethoxyacetophenone and **3a** were used as reactants to give **4m**. Yield: 76%, mp: 154.4–155.2 °C. ^1^H-NMR (CDCl_3_) δ 8.33 (d, *J* = 14.9 Hz, 1 H), 7.83–7.75 (m, 3 H), 7.59 (d, *J* = 2.0 Hz, 1 H), 7.33–7.20 (m, 4 H), 7.08–7.03 (m, 1 H), 6.94 (t, *J* = 7.6 Hz, 1 H), 6.87 (d, *J* = 8.5 Hz, 1H), 6.74 (t, *J* = 6.9 Hz, 1 H), 5.53 (s, 2 H), 3.91 (d, *J* = 2.8 Hz, 6 H). ^13^C-NMR (CDCl_3_) δ 187.2, 160.1 (d), 153.9, 149.4, 148.9, 143.3, 135.8, 130.6, 130.1 (d), 128.5, 128.1 (d), 127.1, 124.8 (d), 124.3, 123.9, 123.7, 122.8 (d), 120.3, 115.8 (d), 110.7, 110.2, 110.1, 56.2, 56.1, 41.1 (d). HRMS (ESI): calcd for C_25_H_21_FN_2_O_3_ [M + H]^+^ 417.1609, found 417.1607. HPLC purity: 99.2%.

*(E)-3-(1-(4-Methylbenzyl)-1H-benzo[d]imidazol-2-yl)-1-(3,4,5-trimethoxyphenyl)prop-2-en-1-one* (**4n**). 3′,4′,5′-Trimethoxyacetophenone and **3b** were used as reactants to give **4n**. Yield: 86%, mp: 190.7–191.8 °C. ^1^H-NMR (CDCl_3_) δ 8.33 (d, *J* = 15.0 Hz, 1 H), 7.86 (t, *J* = 10.7 Hz, 2 H), 7.39–7.29 (m, 5 H), 7.11 (d, *J* = 7.9 Hz, 2 H), 7.02 (d, *J* = 7.9 Hz, 2 H), 5.52 (s, 2 H), 3.95 (s, 9 H), 2.30 (s, 3 H). ^13^C-NMR (CDCl_3_) δ 187.7, 153.3, 148.6, 143.3, 143.1, 138.0, 136.0, 132.7, 129.8, 128.1, 127.9, 126.3, 124.4, 123.7, 120.2, 110.4, 106.4, 61.0, 56.5, 47.0, 21.1. HRMS (ESI): calcd for C_27_H_26_N_2_O_4_ [M + H]^+^ 443.1965, found 443.1961. HPLC purity: 99.6%.

*(E)-3-(1-(2-Fluorobenzyl)-1H-benzo[d]imidazol-2-yl)-1-(3,4,5-trimethoxyphenyl)prop-2-en-1-one* (**4o**). 3′,4′,5′-Trimethoxyacetophenone and **3a** were used as reactants to give **4o**. Yield: 84%, mp: 152.7–153.5 °C. ^1^H-NMR (CDCl_3_) δ 8.36 (d, *J* = 14.9 Hz, 1 H), 7.93–7.84 (m, 2 H), 7.40–7.30 (m, 5 H), 7.14 (dd, *J* = 16.9, 7.9 Hz, 1 H), 7.02 (t, *J* = 8.1 Hz, 1 H), 6.83 (t, *J* = 7.6 Hz, 1 H), 5.61 (s, 2 H), 3.96 (d, *J* = 3.1 Hz, 9 H), 3.89 (d, *J* = 7.4 Hz, 1 H). ^13^C-NMR (CDCl_3_) δ 187.6, 160.1 (d), 153.3, 148.7, 143.2 (d), 135.8, 132.6, 130.1 (d), 128.1, 128.1, 127.8, 124.8 (d), 124.5, 123.8, 122.8 (d), 120.3, 115.8 (d), 110.3, 106.3, 61.0, 56.5, 41.1 (d). HRMS (ESI): calcd for C_26_H_23_FN_2_O_4_ [M + H]^+^ 447.1715, found 447.1719. HPLC purity: 99.5%.

*(E)-1-(4-Ethylphenyl)-3-(1-(4-methylbenzyl)-1H-benzo[d]imidazol-2-yl)prop-2-en-1-one* (**4p**). 4-Ethyl-acetophenone and **3b** were used as reactants to give **4p**. Yield: 84%, mp: 203.6–205.3 °C. ^1^H-NMR (CDCl_3_) δ 8.37 (d, *J* = 15.0 Hz, 1 H), 8.05 (d, *J* = 8.0 Hz, 2 H), 7.90–7.80 (m, 2 H), 7.39–7.27 (m, 5 H), 7.10 (d, *J* = 7.7 Hz, 2 H), 7.01 (d, *J* = 7.8 Hz, 2 H), 5.50 (s, 2 H), 2.73 (q, *J* = 7.5 Hz, 2 H), 2.30 (s, 3 H), 1.28 (t, *J* = 7.6 Hz, 3 H). ^13^C-NMR (CDCl_3_) δ 188.6, 150.6, 148.7, 143.3, 138.0, 136.0, 135.1, 132.7, 129.8, 129.1, 128.5, 128.3, 127.7, 126.3, 124.2, 123.6, 120.3, 110.3, 47.0, 29.0, 21.1, 15.2. HRMS (ESI): calcd for C_26_H_24_N_2_O [M + H]^+^ 381.1961, found 381.1968. HPLC purity: 97.1%.

*(E)-1-(4-Ethylphenyl)-3-(1-(2-fluorobenzyl)-1H-benzo[d]imidazol-2-yl)prop-2-en-1-one* (**4q**). 4-Ethyl-acetophenone and **3a** were used as reactants to give **4q**. Yield: 78%, mp: 165.1–166.3 °C. ^1^H-NMR (CDCl_3_) δ 8.35 (d, *J* = 15.0 Hz, 1 H), 8.00 (d, *J* = 8.3 Hz, 2 H), 7.83–7.78 (m, 2 H), 7.33–7.20 (m, 6 H), 7.08–7.03 (m, 1 H), 6.94 (t, *J* = 7.2 Hz, 1 H), 6.73 (t, *J* = 7.6 Hz, 1 H), 5.53 (s, 2 H), 2.67 (q, *J* = 7.6 Hz, 2 H), 1.21 (t, *J* = 7.6 Hz, 3 H). ^13^C-NMR (CDCl_3_) δ 188.5, 160.1 (d), 150.7, 148.8, 143.3, 135.8, 135.1, 130.1 (d), 129.1, 128.7, 128.3, 128.0 (d), 127.4, 124.8 (d), 124.4, 123.7, 122.8 (d), 120.4, 115.8 (d), 110.2, 41.1 (d), 29.1, 15.2. HRMS (ESI): calcd for C_25_H_21_FN_2_O [M + H]^+^ 385.1771, found 385.1779. HPLC purity: 95.1%.

*(E)-1-(4-Isopropylphenyl)-3-(1-(4-methylbenzyl)-1H-benzo[d]imidazol-2-yl)prop-2-en-1-one* (**4r**). 1-[4-(1-methylethyl)phenyl]ethanone and **3b** were used as reactants to give **4r**. Yield: 83%, mp: 204.7–206.2 °C. ^1^H-NMR (CDCl_3_) δ 8.37 (d, *J* = 15.0 Hz, 1 H), 8.06 (d, *J* = 8.3 Hz, 2 H), 7.88–7.82 (m, 2 H), 7.37–7.27 (m, 5 H), 7.10 (d, *J* = 8.0 Hz, 2 H), 7.01 (d, *J* = 8.0 Hz, 2 H), 5.50 (s, 2 H), 3.04–2.94 (m, 1 H), 2.30 (s, 3 H), 1.29 (d, *J* = 6.9 Hz, 6 H). ^13^C-NMR (101 MHz, CDCl_3_) δ 188.5, 155.2, 148.8, 143.3, 138.0, 136.0, 135.2, 132.7, 129.8, 129.1, 128.5, 127.7, 126.9, 126.3, 124.2, 123.6, 120.3, 110.3, 47.0, 34.4, 23.7, 21.1. HRMS (ESI): calcd for C_27_H_26_N_2_O [M + H]^+^ 395.2118, found 395.2115. HPLC purity: 99.2%.

*(E)-3-(1-(2-Fluorobenzyl)-1H-benzo[d]imidazol-2-yl)-1-(4-isopropylphenyl)prop-2-en-1-one* (**4s**). 1-[4-(1-methylethyl)phenyl]ethanone and **3a** were used as reactants to give **4s**. Yield: 72%, mp: 166.5–167.8 °C. ^1^H-NMR (CDCl_3_) δ 8.41 (d, *J* = 15.0 Hz, 1 H), 8.08 (d, *J* = 8.4 Hz, 2 H), 7.90–7.85 (m, 2 H), 7.39–7.27 (m, 6 H), 7.15–7.10 (m, 1 H), 7.01 (t, *J* = 7.6 Hz, 1 H), 6.80 (t, *J* = 8.3 Hz, 1 H), 5.60 (s, 2 H), 3.04–2.96 (m, 1 H), 1.29 (d, *J* = 6.9 Hz, 6 H). ^13^C-NMR (CDCl_3_) δ 188.5, 160.1 (d), 155.2, 148.8, 143.3, 135.8, 135.2, 130.0 (d), 129.2, 128.7, 128.0 (d), 127.4, 126.9, 124.8 (d), 124.4, 123.7, 122.8 (d), 120.4, 115.8 (d), 110.2, 41.1 (d), 34.5, 23.7. HRMS (ESI): calcd for C_26_H_23_FN_2_O [M + H]^+^ 399.1867, found 399.1862. HPLC purity: 97.8%.

*(E)-4-(3-(1-(4-Methylbenzyl)-1H-benzo[d]imidazol-2-yl)acryloyl)benzonitrile* (**4t**). 4-Acetylbenzonitrile and **3b** were used as reactants to give **4t**. Yield: 82%, mp: 208.6–209.7 °C. ^1^H-NMR (CDCl_3_) δ 8.33 (d, *J* = 15.0 Hz, 1 H), 8.18 (d, *J* = 8.2 Hz, 2 H), 7.91–7.79 (m, 4 H), 7.36 (d, *J* = 7.2 Hz, 3 H), 7.12 (d, *J* = 7.9 Hz, 2 H), 7.00 (d, *J* = 7.9 Hz, 2 H), 5.52 (s, 2 H), 2.30 (s, 3 H). ^13^C-NMR (CDCl_3_) δ 187.7, 148.0, 143.2, 140.5, 138.2, 136.1, 132.6, 132.5, 129.8, 129.5, 129.1, 127.1, 126.2, 124.8, 123.9, 120.5, 117.9, 116.6, 110.5, 47.0, 21.1. HRMS (ESI): calcd for C_25_H_19_N_3_O [M + H]^+^ 378.1601, found 378.1606. HPLC purity: 97.6%.

*(E)-4-(3-(1-(2-Fluorobenzyl)-1H-benzo[d]imidazol-2-yl)acryloyl) benzonitrile* (**4u**). 4-Acetylbenzonitrile and **3a** were used as reactants to give **4u**. Yield: 79%, mp: 167.5–169.4 °C. ^1^H-NMR (CDCl_3_) δ 8.34 (d, *J* = 14.9 Hz, 1 H), 8.20 (d, *J* = 8.4 Hz, 2 H), 7.93 (d, *J* = 14.9 Hz, 1 H), 7.86 (d, *J* = 6.7 Hz, 1 H), 7.82 (d, *J* = 8.4 Hz, 2 H), 7.41–7.30 (m, 4 H), 7.16–7.11 (m, 1 H), 7.03 (t, *J* = 7.4 Hz, 1 H), 6.82 (t, *J* = 7.5 Hz, 1 H), 5.61 (s, 2 H). ^13^C-NMR (CDCl_3_) δ 187.7, 160.1 (d), 148.1, 143.3, 140.4, 135.9, 132.6, 130.2 (d), 129.3, 129.1, 128.1 (d), 127.2, 124.9, 124.8 (d), 124.0, 122.7 (d), 120.6, 117.9, 116.6, 115.9 (d), 110.3, 41.2 (d). HRMS (ESI): calcd for C_24_H_16_FN_3_O [M + H]^+^ 382.1350, found 382.1352. HPLC purity: 95.4%.

### 3.2. Biology

#### 3.2.1. Topo II Mediated DNA Relaxation Assay In Vitro

We used Topo II assay kit (TopoGEN, Buena Vista, CO, USA) to study the effects of compounds on Topo II-catalyzed DNA relaxation according to the kit instructions with minor modifications. Relaxation assays were performed in a final volume of 20 μL in Topo II reaction buffer (50 mM Tris-HCl, pH 8.0, 150 mM NaCl, 10 mM MgCl_2_, 2 mM ATP, 0.5 mM DTT, and 30 μg/mL BSA) with 0.2 μg of pBR322 DNA (TaKaRa, Shiga, Japan). Then compounds and 1 U human Topo II were added to the reaction at a constant solvent volume. Etoposide (100 μM) was used as a comparative control. The reaction was incubated for 30 min at 37 °C. The reaction was stopped with 5× stop buffer (5 μL per 20 μL reaction volume). 5× stop buffer contained 5% sarcosyl, 0.02% bromophenol blue, and 25% glycerol. The mixtures were applied to 1% agarose gel and subjected to electrophoresis for 1.5 h at 75 V, in TAE buffer (40 mM Tris-acetate, 2 mM EDTA). Gels were stained for 30 min in an aqueous solution of Ged Red (0.5 μg/mL). DNA bands were visualized by UV light transillumination and then photographed with an Alpha Innotech digital imaging system (Boston Microscopes, Boston, MA, USA). Semi-quantification was performed on western blot gel photographs by using software ImageJ 1.53a, with positive control (lane D: pBR322 DNA) as 100%, negative control (lane T: pBR322 DNA + Topo II) as 0% and comparative control (lane E: pBR322 DNA + Topo II + etoposide 100 μM).

#### 3.2.2. Topo II Mediated DNA Cleavage Assay

The mixture of Topo II (6 units), 0.1 μg of supercoiled pBR322 DNA, and 50 μM compounds (or 100 μM etoposide) were performed in a final volume of 20 μL in Topo II reaction buffer (50 mM Tris-HCl, pH 8.0, 150 mM NaCl, 10 mM MgCl_2_, 2 mM ATP, 0.5 mM DTT, and 30 μg/mL BSA). After incubation at 37 °C for 6 min to achieve cleavage relegation equilibrium, the cleavage intermediates were captured by the addition of 2 μL of 1% SDS followed by the addition of 2 μL of 250 mM Na_4_EDTA at pH 8.0. Proteinase K (2 μL, 0.8 mg/mL) was added and the reactions were incubated at 45 °C for 30 min to digest Topo II. The sample was mixed with 5 μL of agarose gel loading buffer (30% sucrose, 0.5% bromophenol blue and 0.5% xylene cyano FF in 10 mM Tris-HCl, pH 7.9) and heated at 72 °C for 2 min. Electrophoresis was carried out in a 1% agarose gel in TAE buffer (40 mM Tris-acetate and 2 mM EDTA) at 75 V for 1 h. The gel was stained in Ged Red aqueous solution (0.5 μg/mL) for 30 min and kept on electrophoresis at 75 V for 30–45 min. Cleavage was monitored by the conversion of negatively supercoiled plasmid to nicked DNA. DNA bands were visualized by UV light and photographed with an Alpha Innotech digital imaging system. Semi-quantification was performed on western blot gel photographs by using software ImageJ 1.53a.

#### 3.2.3. DNA Unwinding Assay

The relaxed pBR322 DNA used for this unwinding assay was previously relaxed with Topo I in the reaction buffer (50 mM Tris-HCl, pH = 7.5, 50 mM KCl, 10 mM MgCl_2_, 0.5 mM DTT, 0.1 mM EDTA, and 30 μg/mL BSA). Then other components were added. Assay mixtures contained 0.1 μg of relaxed pBR322 plasmid DNA, Topo I (1 units), and compounds in 20 μL of Topo I reaction buffer. After incubating the DNA and drug for 10 min at room temperature, Topo I was added, and the reaction was incubated at 37 °C for 30 min. The sample was mixed with 5 μL of agarose gel loading buffer (30% sucrose, 0.5% bromophenol blue and 0.5% xylene cyano FF in 10 mM Tris-HCl, pH 7.9). Samples were subjected to electrophoresis in 1 × TAE buffer (40 mM Tris-acetate, 2 mM EDTA). DNA bands were visualized by transillumination with UV light and then photographed with an Alpha Innotech digital imaging system (Boston Microscopes).

#### 3.2.4. In Vitro Anti-Proliferation Assay

MTT assay was used to measure the growth inhibitory effects of **4a**–**4u** in various cancer cell lines. HepG2, A549, LNCaP, MG63 cells obtained from National Infrastructure of Cell Line Resource (Beijing, China). HepG2, LNCaP and MG63 cells were cultured with DMEM medium supplemented with 10% FBS, while A549 were with RPMI-1640 medium. Each cell line was plated at a density of 5000 per well in 96-well microplates, incubated at 37 °C in a humidified incubator with 5% CO_2_ for 24 h. Compounds **4a**–**4u** were added to the wells at increasing concentrations (0–50 μM). After 48 h, the medium was replaced with new medium (100 μL, containing 50 μg MTT) in each well. The cells were further incubated at 37 °C for 4 h. At the end of the incubation, the untransformed MTT was removed, and 100 μL of DMSO was added. The microplates were well shaken to dissolve the formazan dye, and the absorbance at 570 nm was measured using a microplate reader (Bio-Tek). The amount of absorbance (A570) is proportional to the cell numbers. The inhibition rates of different concentrations of test compounds were calculated with the following equation. The IC_50_ values of test compounds were calculated after nonlinear regression of logarithmic concentrations and cell proliferation inhibition rates:

$$\mathrm{Cell}\;\mathrm{proliferation}\;\mathrm{inhibition}\;\mathrm{rate}\;\%=\frac{\left(A570_{control}-A570_{sample}\right)\times 100}{A570_{control}}\%$$

#### 3.2.5. Clonogenic Assay

To determine the population dependence and proliferative capacity of A549 cells after treatment of compounds, the clonogenic assay was implemented on the basis of the method previously reported with minor changes. A549 cells were plated at a density of 700 per well in six-well plates and grown at 37 °C for 24 h and then treated with increasing concentrations of compound (0–3 μM) for another 24 h, subsequently, medium was replaced with fresh medium and incubated for two weeks. The colonies developed were fixed with methanol and stained using 0.4% trypan blue. The number of colonies in each plate was photographed with a digital camera and then counted with ImageJ 1.53a.

#### 3.2.6. Cell Migration Assay

Approximately 3 × 10^5^ A549 cells per well were cultured in a six-well plate to 80% confluence. Wound generation was performed using a 10 μL pipet tip across the center of the well and each well was washed twice with PBS to remove the floating/detached cells. After, the cells were exposed to each of **4d** and **4n** (1 and 2 μM) for 24 h and were allowed to migrate in the medium (RPMI-1640 with 2% FBS). The wound was assessed by a JuLI Intelligent fluorescent cell monitor (NanoEnTek, Seoul, South Korea) at different time points (0, 24, 48 and 72 h).

#### 3.2.7. DAPI Staining

Apoptotic cell death induced by **4d** or **4n** was examined by fluorescence microscopy using DAPI staining. Briefly, A549 cells were seeded to six-well plate in a density of 8 × 10^4^ per well and incubated with or without the indicated concentration of **4d** or **4n** for 48 h. Then DAPI (1 μg/mL final concentration) was added and incubated for 10 min. After one wash with PBS, cells were photo-graphed using a Olympus CX31 Fluorescence Microscope (Olympus, Shinjuku, Japan). Apoptotic cells were identified by morphologic alterations (nuclear condensation and cell fragmentation). The photographic analyses and quantification of apoptotic cells were performed by using software ImageJ 1.53a. NAF was calculated by the product of average nuclear area and circularity.

## 4. Conclusions

In conclusion, a novel series of benzimidazole-chalcone hybrids were designed and synthesized as potential Topo II-targeting anticancer agents. Most of the tested compounds displayed strong inhibitory activity against the catalytic function of Topo II. Mechanistic studies indicated that **4d** and **4n** act as non-intercalative Topo II catalytic inhibitors. The BCHs displayed favorable antiproliferative activities with low micromolar IC_50_ values toward the four tested cancer cell lines, especially on A549 cells. Hybrid **4d** and **4H** not only showed excellent anti-proliferative activity with respective IC_50_ values of 3.6, 3.8 μM on A549 cells, but also inhibited A549 cell migration and probably induced cancer cell apoptosis. The mode of action postulated by the molecular docking suggests a possible occupation of the ATP binding pocket of Topo II by **4d** and **4n**. This research identified novel benzimidazole-chalcone hybrids and diversified the scope of Topo II inhibitors, which would provide valuable foundation for the research on anticancer drugs. Ideally further research on pharmaceutical kinetics and toxicity of the optimized compounds may forward one step further to the discovery of a novel chemotherapeutic drug.

## Figures and Tables

**Table 1:** Inhibition of cancer cells proliferation for compounds **4a**–**4u**.

Compound	IC_50_ (Mean ± SD) (μM) ^a^				Topo II Inhibition	
	A549	HePG2	MG63	LNCaP	Inhibition Rate (%) ^b^	Indicator
**4a**	2.8 ± 0.2	4.0 ± 0.6	4.0 ± 0.5	3.5 ± 0.1	28.3 ± 0.7	+
**4b**	3.6 ± 0.6	4.6 ± 0.9	5.5 ± 0.8	4.2 ± 0.2	67.1 ± 2.1	+
**4c**	8.3 ± 2.8	6.9 ± 1.1	6.7 ± 0.6	7.7 ± 0.4	75.5 ± 2.6	++
**4d**	3.6 ± 0.7	4.5 ± 1.0	4.7 ± 0.8	5.4 ± 0.1	86.9 ± 2.7	++
**4e**	5.5 ± 1.5	5.5 ± 1.7	6.7 ± 0.6	8.6 ± 0.5	54.8 ± 1.0	+
**4f**	6.1 ± 4.9	10.8 ± 5.7	5.5 ± 0.5	8.5 ± 1.5	44.2 ± 0.3	+
**4g**	3.3 ± 0.6	4.4 ± 1.3	4.7 ± 0.5	3.6 ± 0.2	90.2 ± 2.1	++
**4h**	2.9 ± 0.2	4.6 ± 0.8	4.4 ± 0.1	3.6 ± 0.1	−0.9 ± 0.2	-
**4i**	5.6 ± 2.7	4.9 ± 1.5	5.7 ± 0.6	6.1 ± 0.2	93.9 ± 1.4	++
**4j**	3.1 ± 0.1	4.4 ± 0.9	5.7 ± 0.8	6.1 ± 0.1	38.5 ± 1.0	+
**4k**	8.8 ± 1.0	7.0 ± 0.5	10.7 ± 0.6	15.7 ± 2.7	59.2 ± 0.9	+
**4l**	3.7 ± 1.1	5.1 ± 0.3	10.3 ± 0.6	3.4 ± 0.2	65.8 ± 1.3	+
**4m**	3.1 ± 0.7	3.9 ± 0.3	5.0 ± 0.4	3.5 ± 0.4	n.d. ^[c]^	n.d. ^[c]^
**4n**	3.8 ± 1.8	4.6 ± 0.2	4.1 ± 0.6	3.6 ± 0.3	94.9 ± 2.9	++
**4o**	3.4 ± 0.4	4.5 ± 0.2	6.2 ± 0.6	3.4 ± 0.5	97.4 ± 1.6	++
**4p**	5.7 ± 0.8	6.5 ± 0.3	3.7 ± 0.6	5.7 ± 0.5	108.6 ± 1.0	++
**4q**	4.3 ± 1.0	5.5 ± 0.3	4.1 ± 0.9	4.3 ± 1.4	105.2 ± 0.6	++
**4r**	5.8 ± 0.7	6.5 ± 0.2	4.3 ± 1.3	5.8 ± 0.3	106.4 ± 0.6	++
**4s**	5.5 ± 1.0	5.6 ± 0.2	3.8 ± 0.8	4.9 ± 0.3	103.1 ± 1.2	++
**4t**	12.1 ± 1.1	22.4 ± 1.8	47.7 ± 1.6	20.1 ± 2.4	41.2 ± 1.5	+
**4u**	8.2 ± 1.6	8.1 ± 0.4	18.5 ± 3.0	18.0 ± 0.5	70.9 ± 0.7	+
**2a**	74.3 ± 2.7	>100	>100	86.4 ± 2.4	23.2 ± 1.6	-
**chalcone**	67.3 ± 1.3	86.4 ± 0.9	>100	52.4 ± 1.1	12.5 ± 2.5	-
**etoposide**	11.8 ± 1.2	5.2 ± 1.3	12.1 ± 1.1	13.3 ± 1.6	73.6 ± 0.6	+

^a^, Each assay was performed in quadruplicate with a number of determinations n > 2, and the results are expressed as mean ± SD, whereas the IC_50_ means the concentration of drug needed to reduce the cell number to 50% of vehicle. ^b^, Topo II inhibition potency of compounds at 20 μM was semi-quantified in the DNA relaxation assay, normalized by positive control (lane D) as 100%, negative control (lane T) as 0%, comparative control (lane E). Indicators were set as follows: -, no obvious inhibition (<25%); +, weak inhibition (≥25%, <75%); ++, strong inhibition (≥75%). ^[c]^ Not determined.

## Supplementary Materials

The following are available online, ^1^H- and ^13^C-NMR spectra of final compounds, detailed docking results of **4d**, **4n** and original ligand with Topo II (1ZXM), Table S1: Total scores of **4n**, **4d** and original ligand of Topo II (1ZXM), Figure S1: Docking model of **4n** with Topo II protein (1ZXM), Figure S2: Docking model of **4d** with Topo II protein (1ZXM), Figure S3: Docking model of Topo II protein (1ZXM)with its original ligand, Figure S4: Docking results of Topo II protein (1ZXM)with its original ligand.

## Abbreviations

Topo II	type II topoisomerase
BCHs	benzimidazole-chalcone hybrids
IC_50_	half maximal inhibitory concentration
ATP	adenosine triphosphate
EB	ethidium bromide
DAPI	4′,6-diamidino-2-phenylindole
ADP	adenosine diphosphate
