# Supplementary material for: Design, Synthesis and Anti-Tumor Activity of Novel Benzimidazole-Chalcone Hybrids as Non-Intercalative Topoisomerase II Catalytic Inhibitors
Source: Molecules. 2020 Jul 12;25(14):3180. doi: 10.3390/molecules25143180 (PMC7397320; doi:10.3390/molecules25143180)

Supplementary Materials

Design, Synthesis and Anti-tumor Activity of Novel Benzimidazole-Chalcone Hybrids as Non-Intercalative Topoisomerase II Catalytic Inhibitors

Wei Zhou [1,*], Wenjin Zhang [1], Yi Peng [1], Zhi-Hong Jiang [2], Lanyue Zhang [1], Zhiyun Du [1,*]

[1] *School of Biomedical and Pharmaceutical Sciences, Guangdong University of Technology, Guangzhou 510006, P.R. China*

[2] *State Key Laboratory of Quality Research in Chinese Medicine, Macau Institute for Applied Research in Medicine and Health, Macau University of Science and Technology, Avenida Wai Long, Taipa, Macau 999078, P.R. China*

* Correspondence: zhou_wei@gdut.edu.cn (W. Z.), zhiyundu@foxmail.com (Z.D.);

Contents of Supplementary Materials

^{1}H NMR and ^{13}C NMR spectra of compound **4a** ········ 3

^{1}H NMR and ^{13}C NMR spectra of compound **4b** ········ 4

^{1}H NMR and ^{13}C NMR spectra of compound **4c** ········ 5

^{1}H NMR and ^{13}C NMR spectra of compound **4d** ········ 6

^{1}H NMR and ^{13}C NMR spectra of compound **4e** ········ 7

^{1}H NMR and ^{13}C NMR spectra of compound **4f** ········ 8

^{1}H NMR and ^{13}C NMR spectra of compound **4g** ········ 9

^{1}H NMR and ^{13}C NMR spectra of compound **4h** ········ 10

^{1}H NMR and ^{13}C NMR spectra of compound **4i** ········ 11

^{1}H NMR and ^{13}C NMR spectra of compound **4j** ········ 12

^{1}H NMR and ^{13}C NMR spectra of compound **4k** ········ 13

^{1}H NMR and ^{13}C NMR spectra of compound **4l** ········ 14

^{1}H NMR and ^{13}C NMR spectra of compound **4m** ········ 15

^{1}H NMR and ^{13}C NMR spectra of compound **4n** ········ 16

^{1}H NMR and ^{13}C NMR spectra of compound **4o** ········ 17

^{1}H NMR and ^{13}C NMR spectra of compound **4p** ········ 18

^{1}H NMR and ^{13}C NMR spectra of compound **4q** ········ 19

^{1}H NMR and ^{13}C NMR spectra of compound **4r** ········ 20

^{1}H NMR and ^{13}C NMR spectra of compound **4s** ········ 21

^{1}H NMR and ^{13}C NMR spectra of compound **4t** ········ 22

^{1}H NMR and ^{13}C NMR spectra of compound **4u** ········ 23

Supplementary Table 1: Total scores of 4n, 4d and original ligand of Topo II (1ZXM) ········ 24

Supplementary Figure 1: Docking model of 4d with Topo II protein (1ZXM) ········ 24

Supplementary Figure 2: Docking model of 4d with Topo II protein (1ZXM) ········ 24

Supplementary Figure 3: Docking model of Topo II protein (1ZXM) with its original ligand ········ 25

Supplementary Figure 4: Docking result of Topo II protein (1ZXM) with its original ligand ········ 25

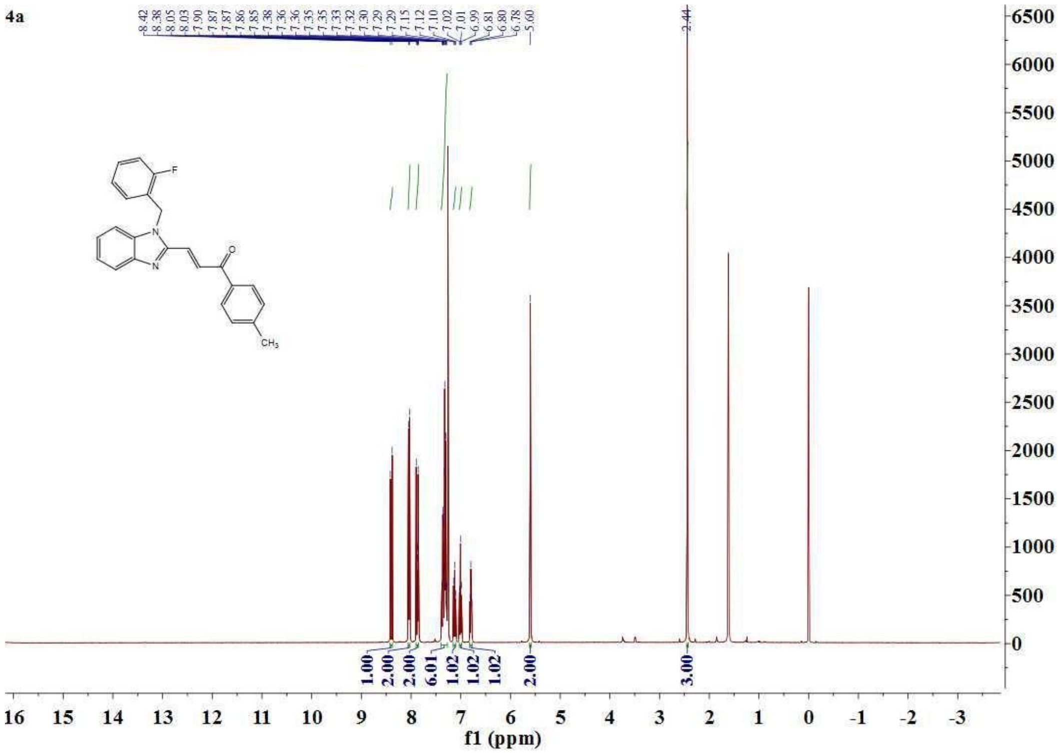

^{1}H NMR (400 MHz, $CDCl_3$) of compound **4a**

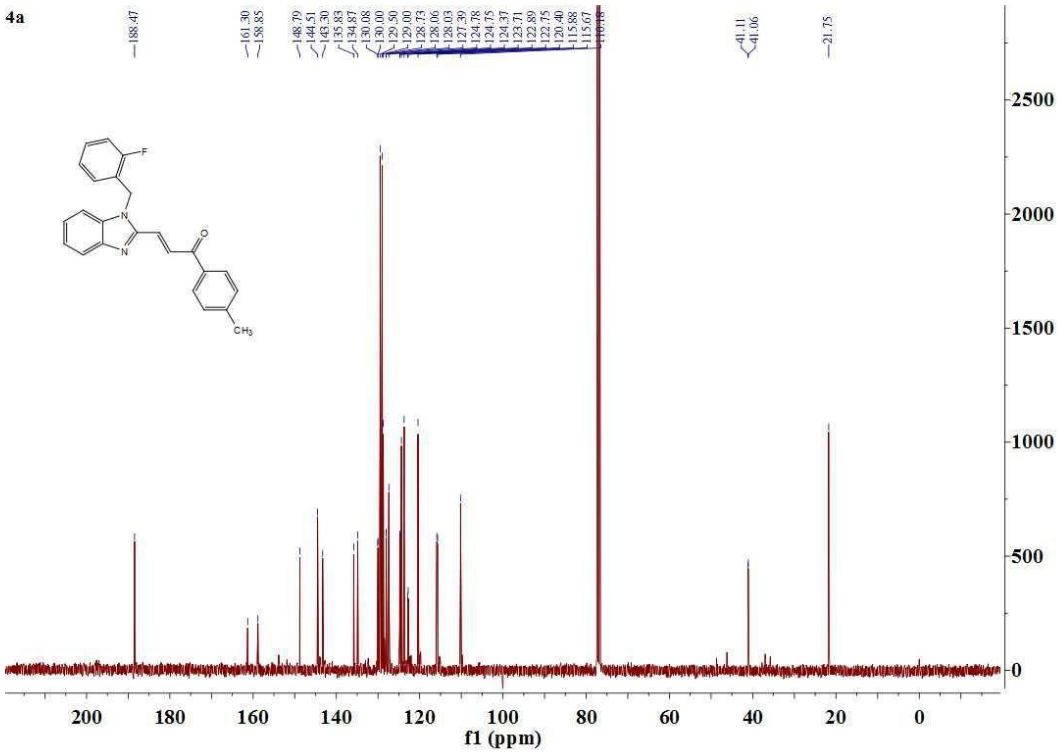

^{13}C NMR (101 MHz, $CDCl_3$) of compound **4a**

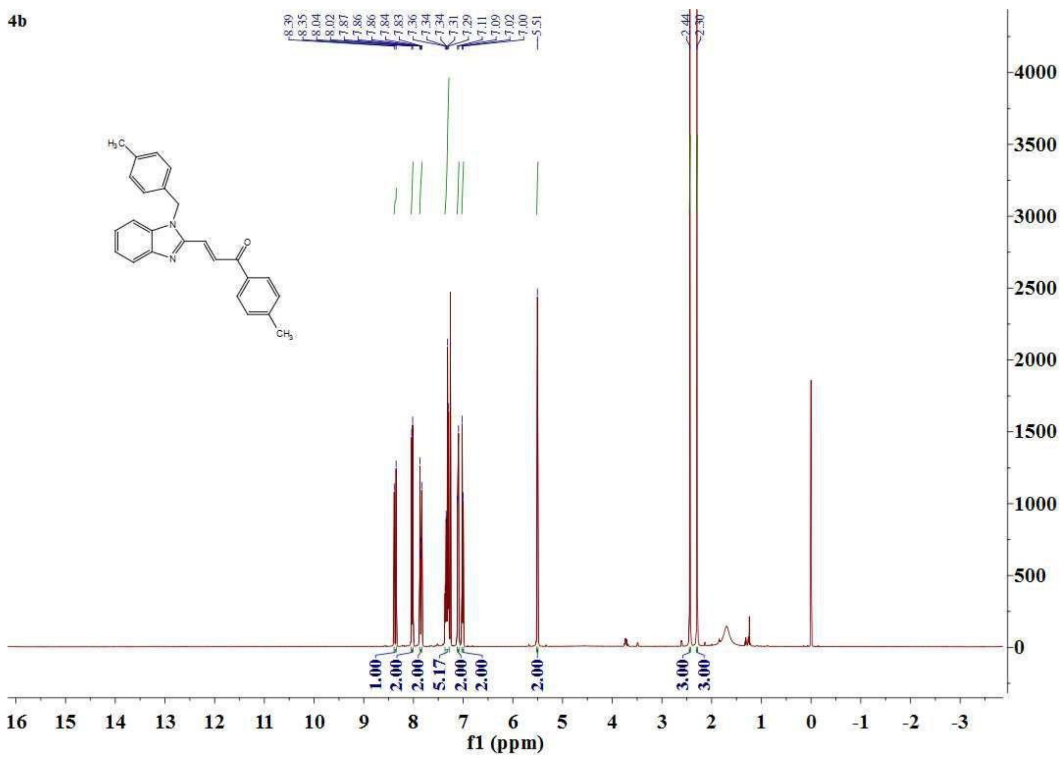

^{1}H NMR (400 MHz, $CDCl_3$) of compound **4b**

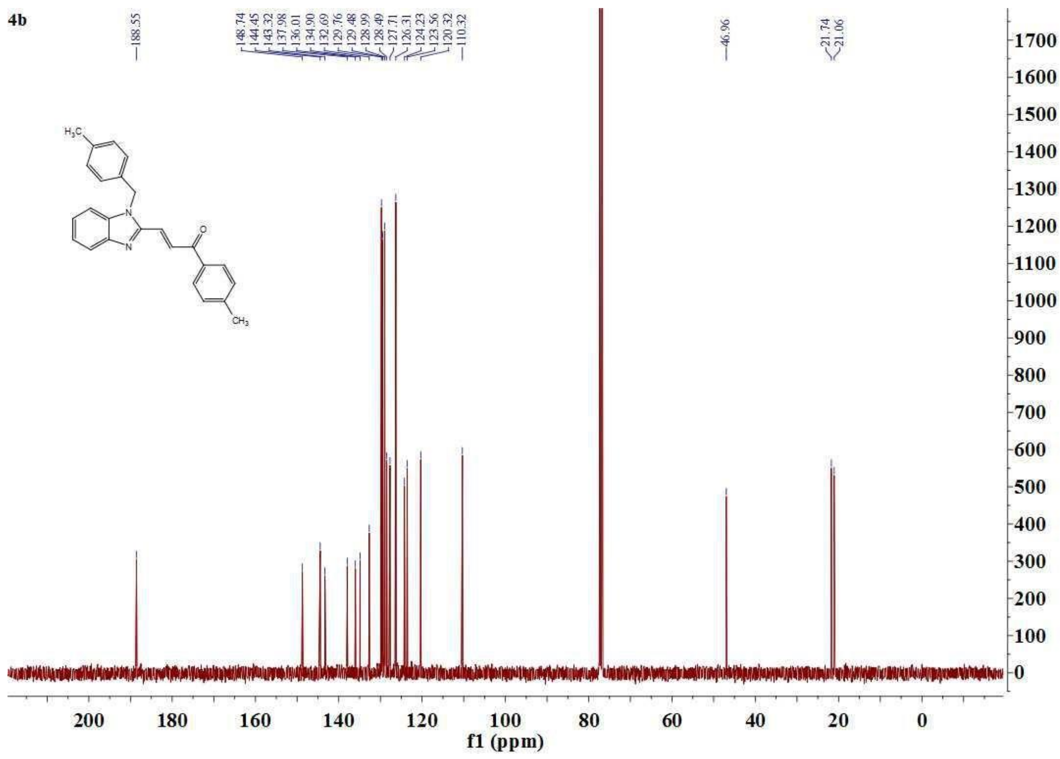

^{13}C NMR (101 MHz, $CDCl_3$) of compound **4b**

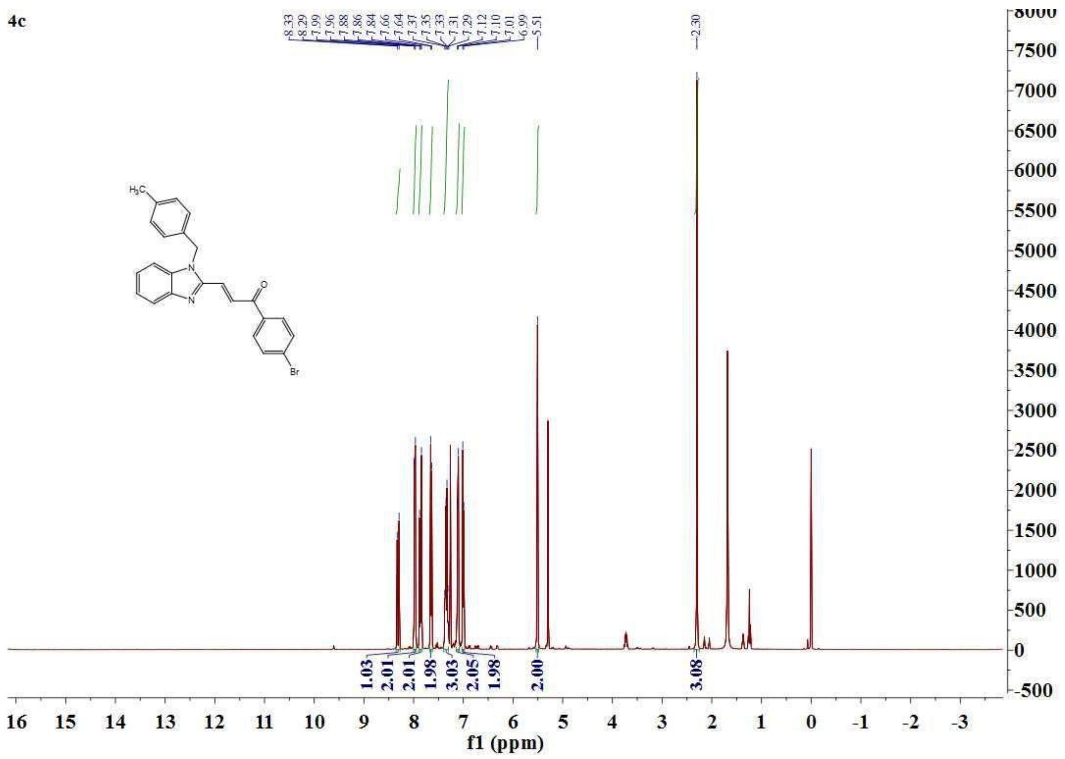

^{1}H NMR (400 MHz, CDCl $_{3}$) of compound **4c**

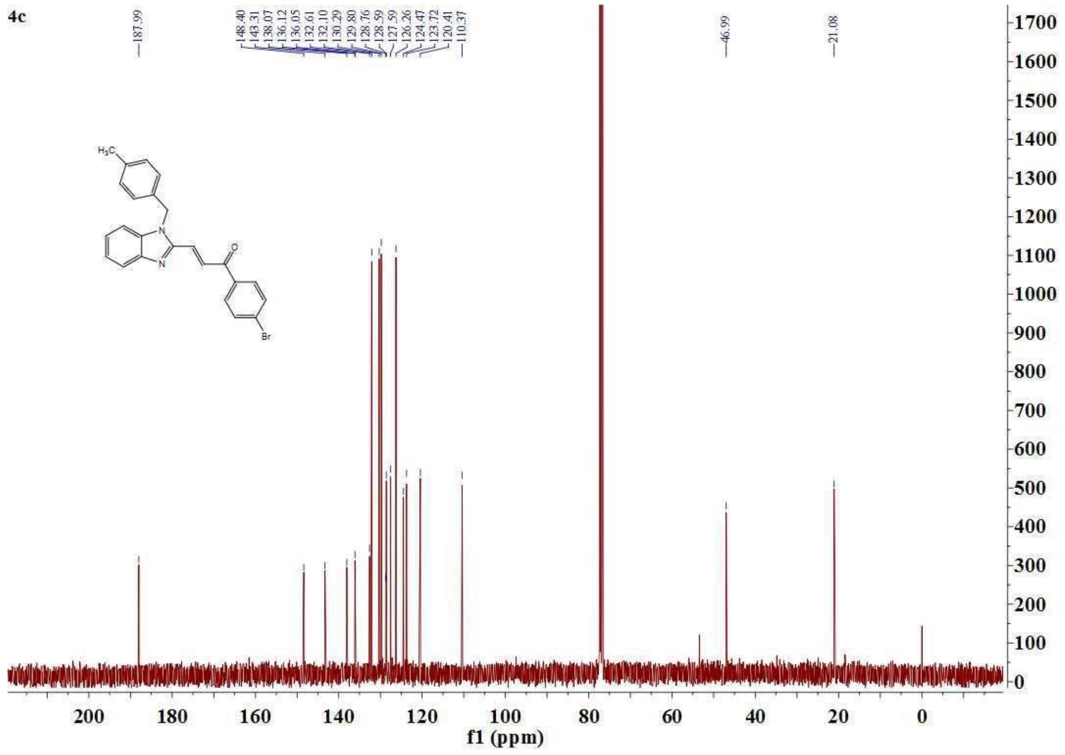

^{13}C NMR (101 MHz, CDCl $_{3}$) of compound **4c**

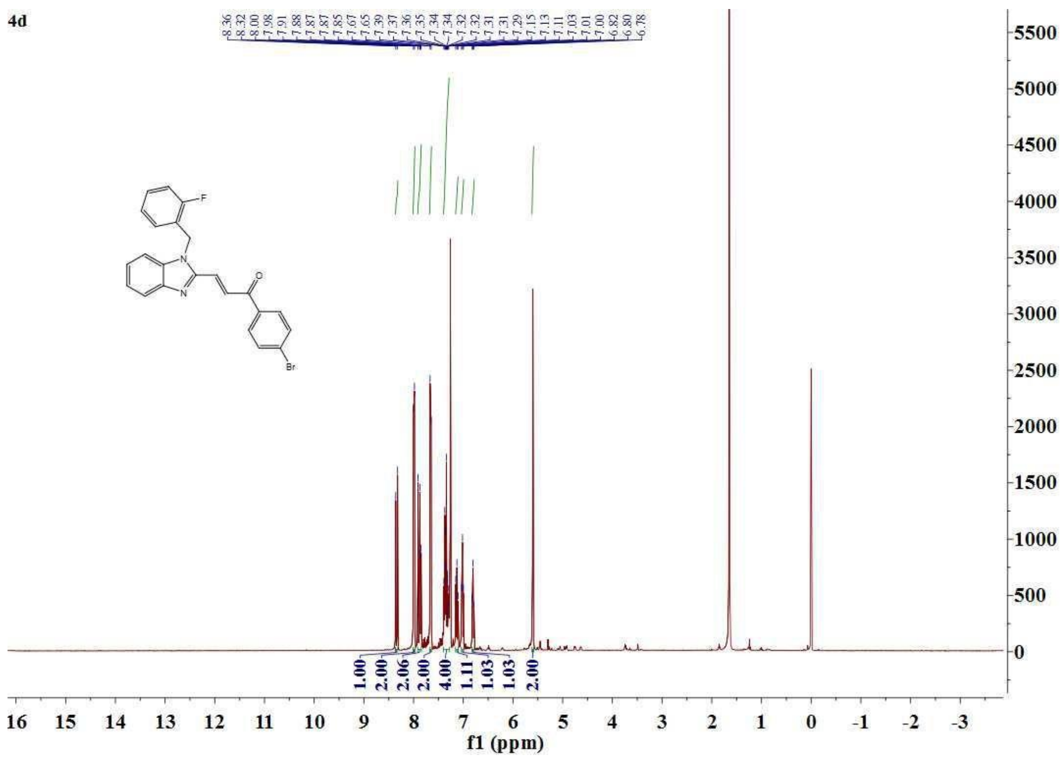

^{1}H NMR (400 MHz, CDCl $_3$) of compound **4d**

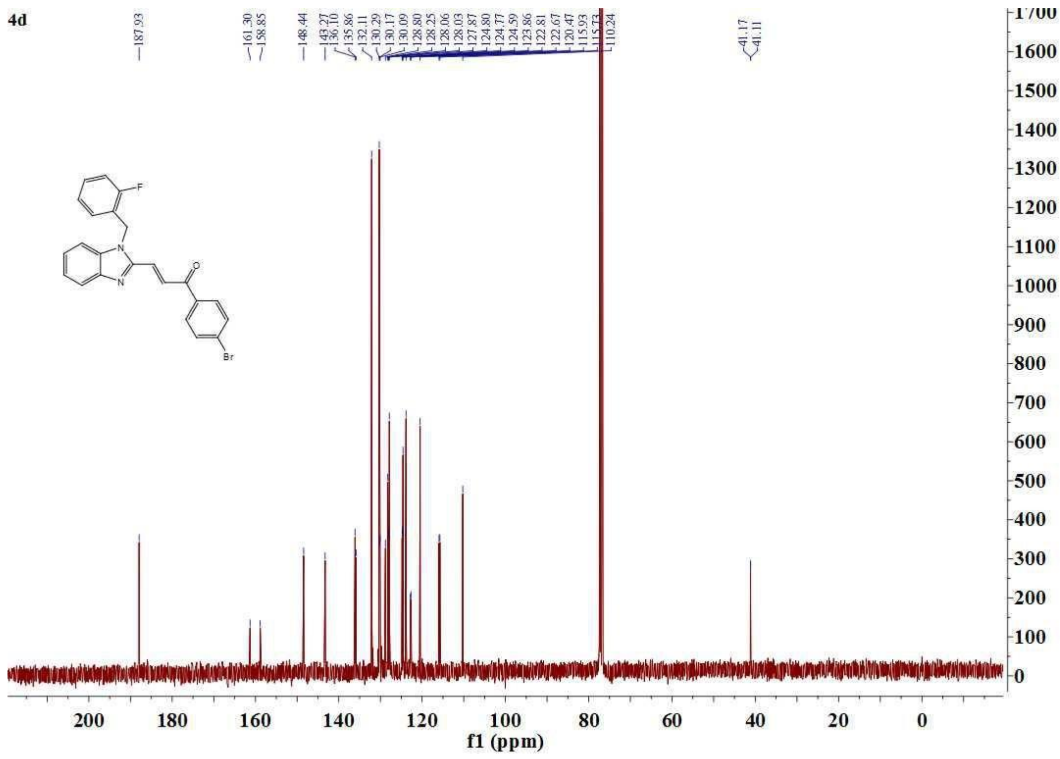

^{13}C NMR (101 MHz, CDCl $_3$) of compound **4d**

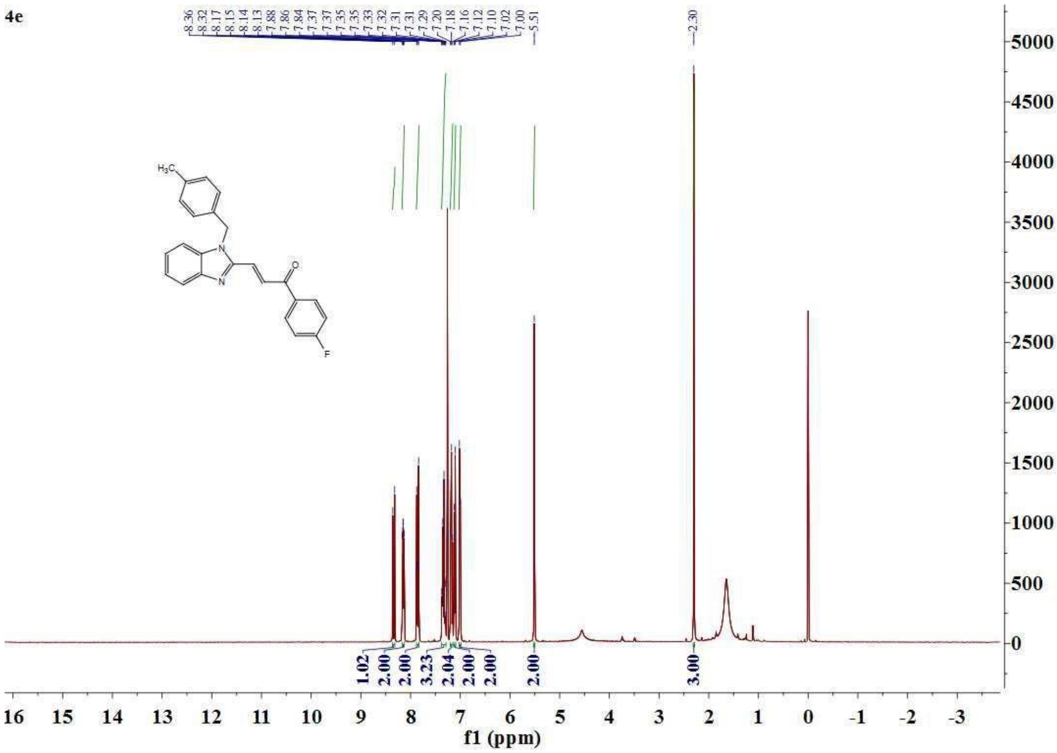

^{1}H NMR (400 MHz, $CDCl_3$) of compound **4e**

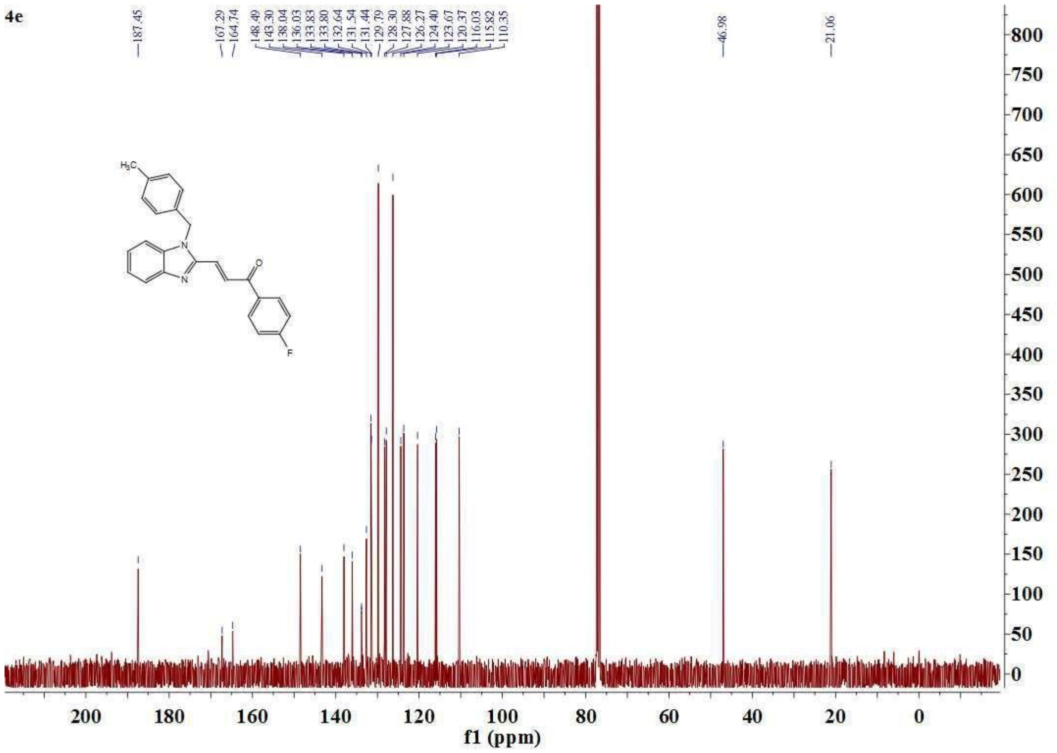

^{13}C NMR (101 MHz, $CDCl_3$) of compound **4e**

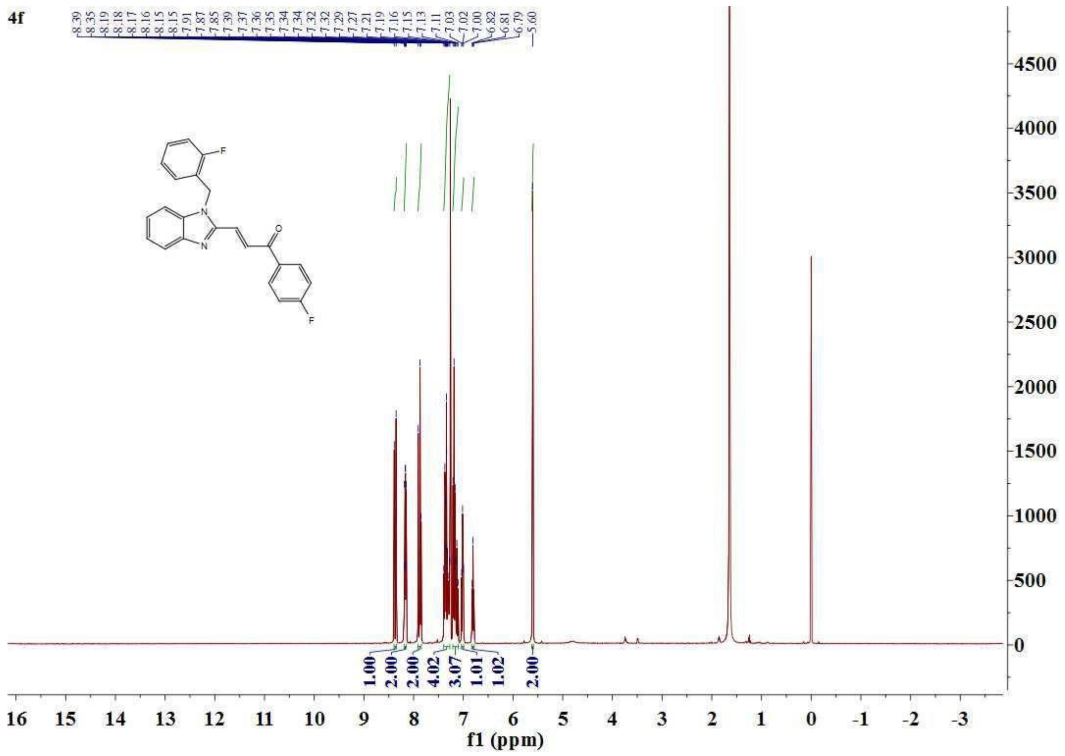

^1H NMR (400 MHz, $CDCl_3$) of compound **4f**

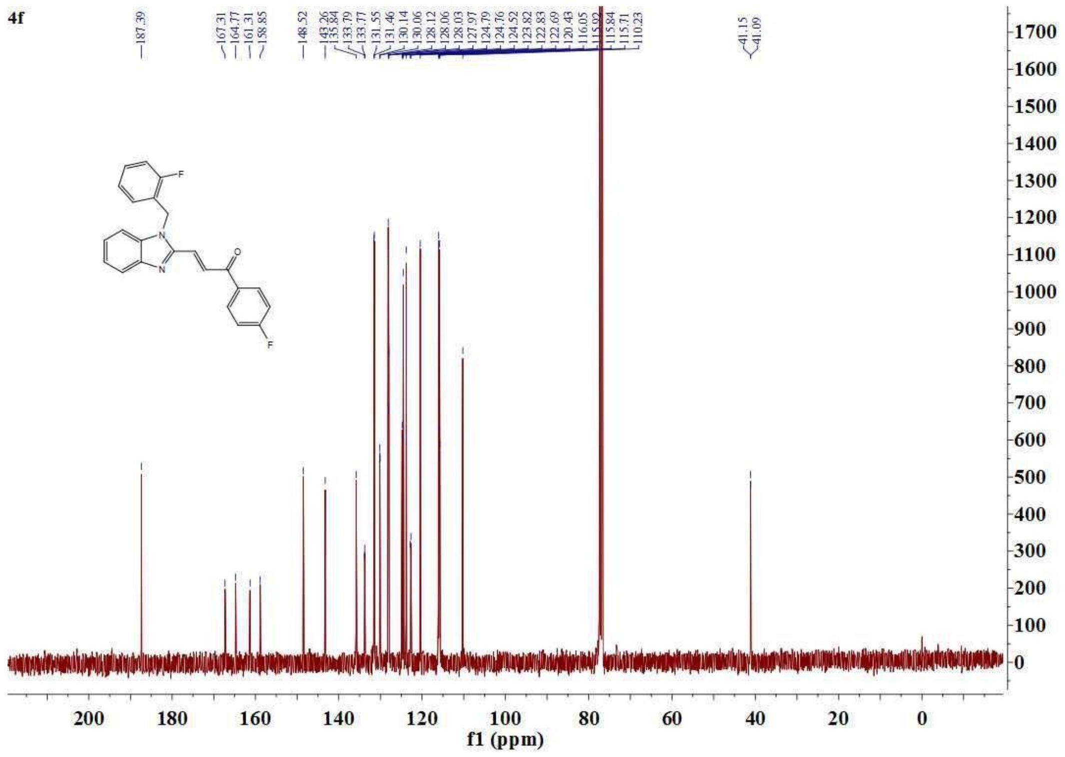

^{13}C NMR (101 MHz, $CDCl_3$) of compound **4f**

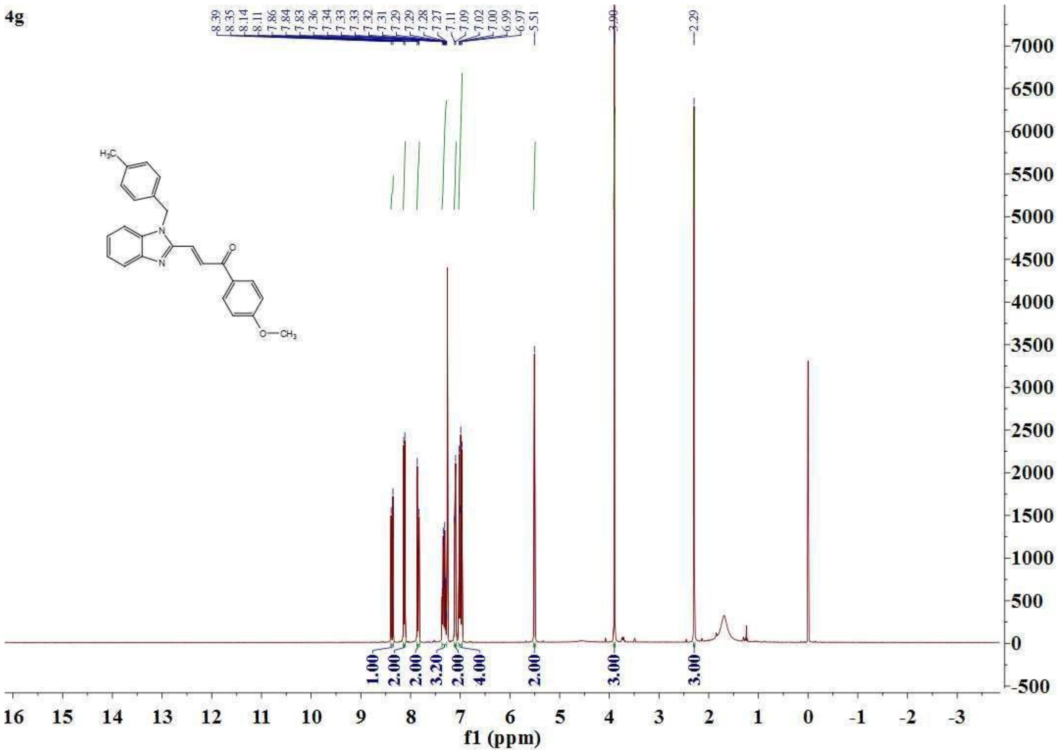

^{1}H NMR (400 MHz, CDCl 3) of compound **4g**

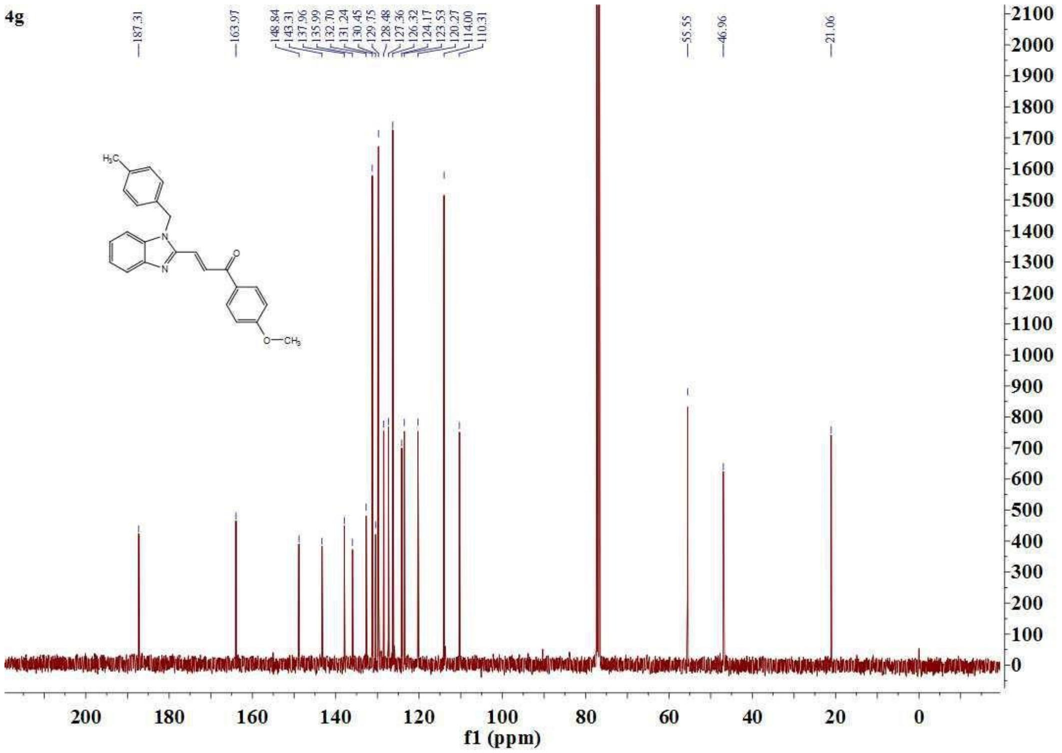

^{13}C NMR (101 MHz, CDCl 3) of compound **4g**

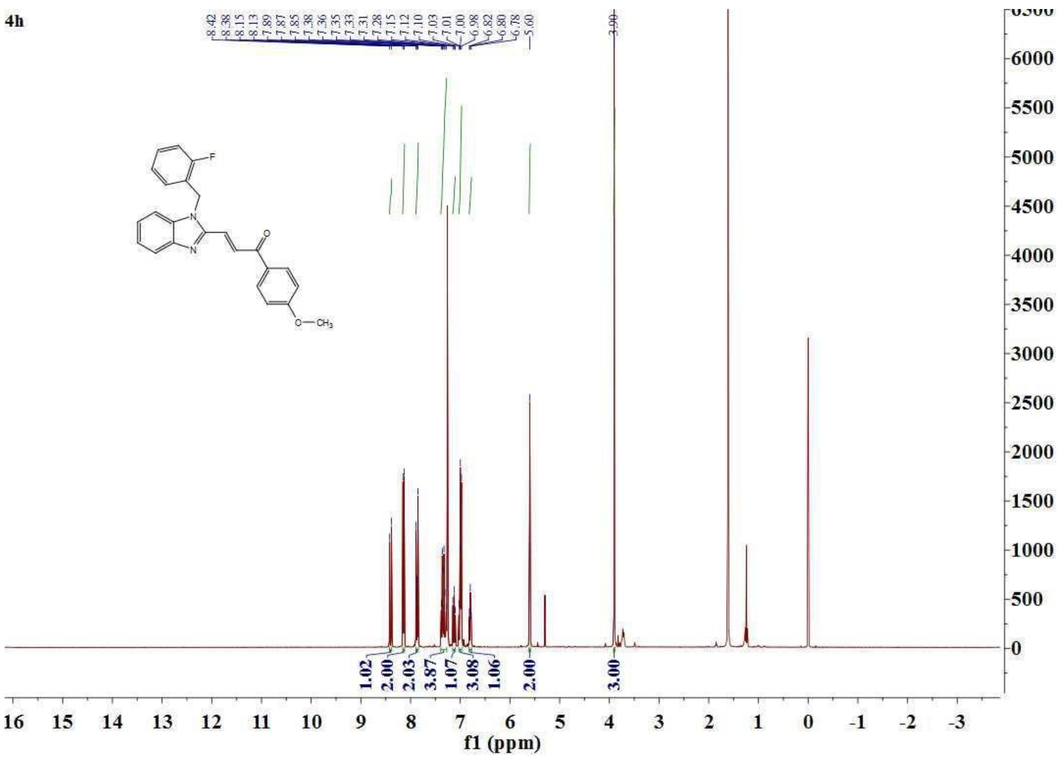

^{1}H NMR (400 MHz, $CDCl_3$) of compound **4h**

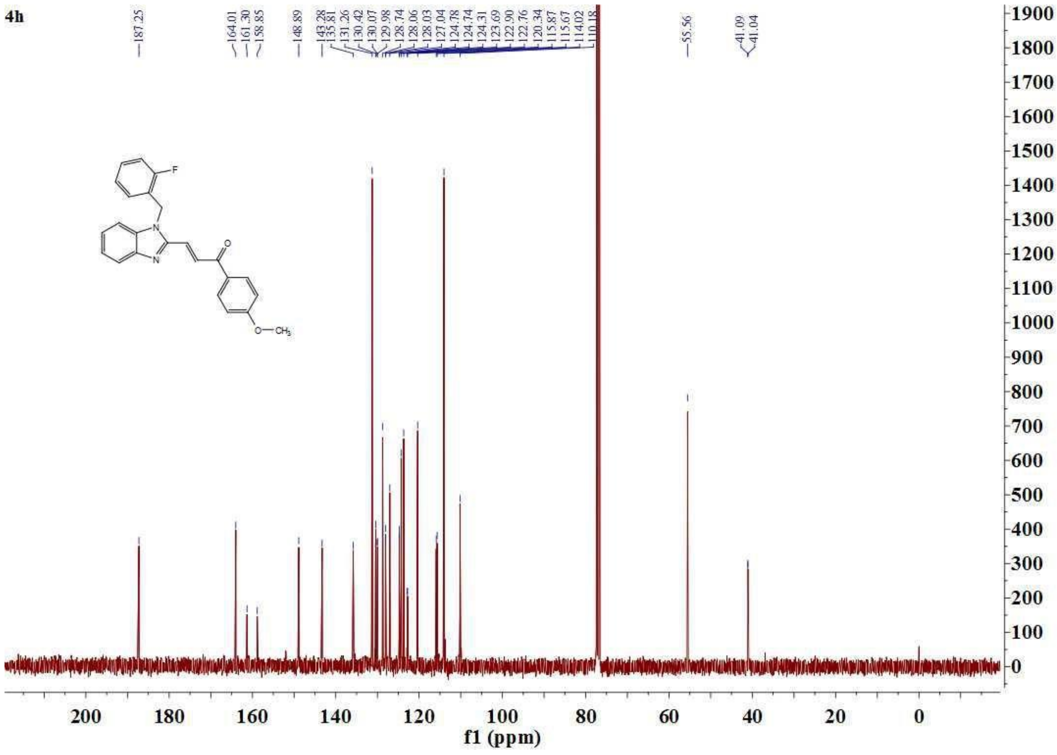

^{13}C NMR (101 MHz, $CDCl_3$) of compound **4h**

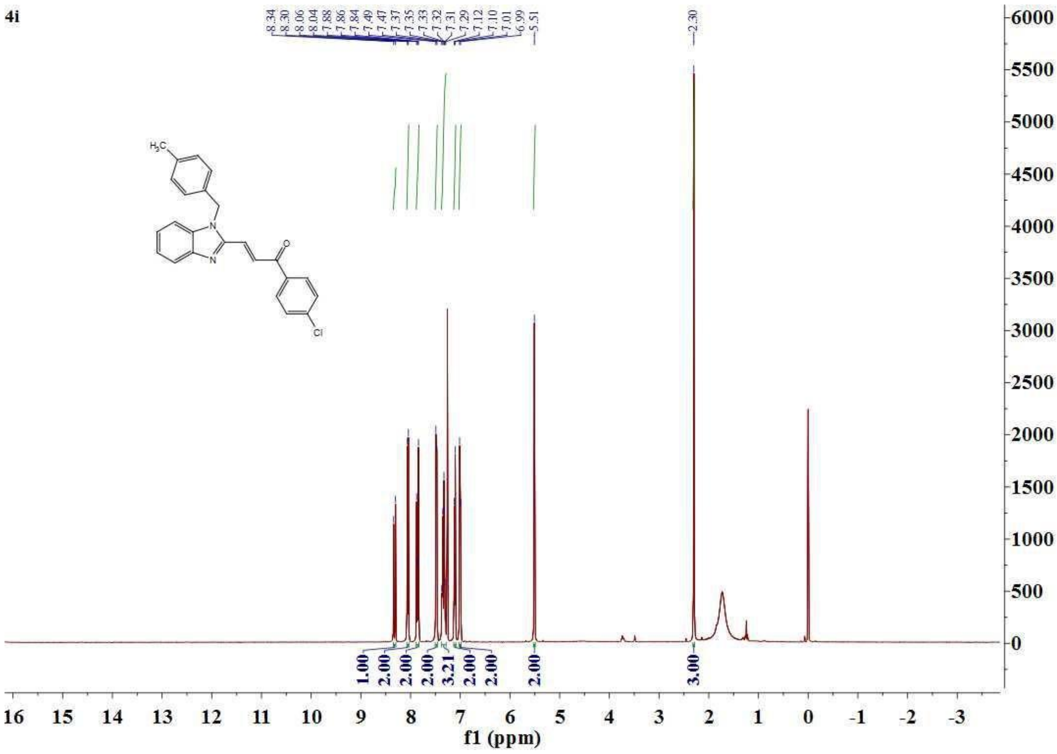

^{1}H NMR (400 MHz, $CDCl_3$) of compound **4i**

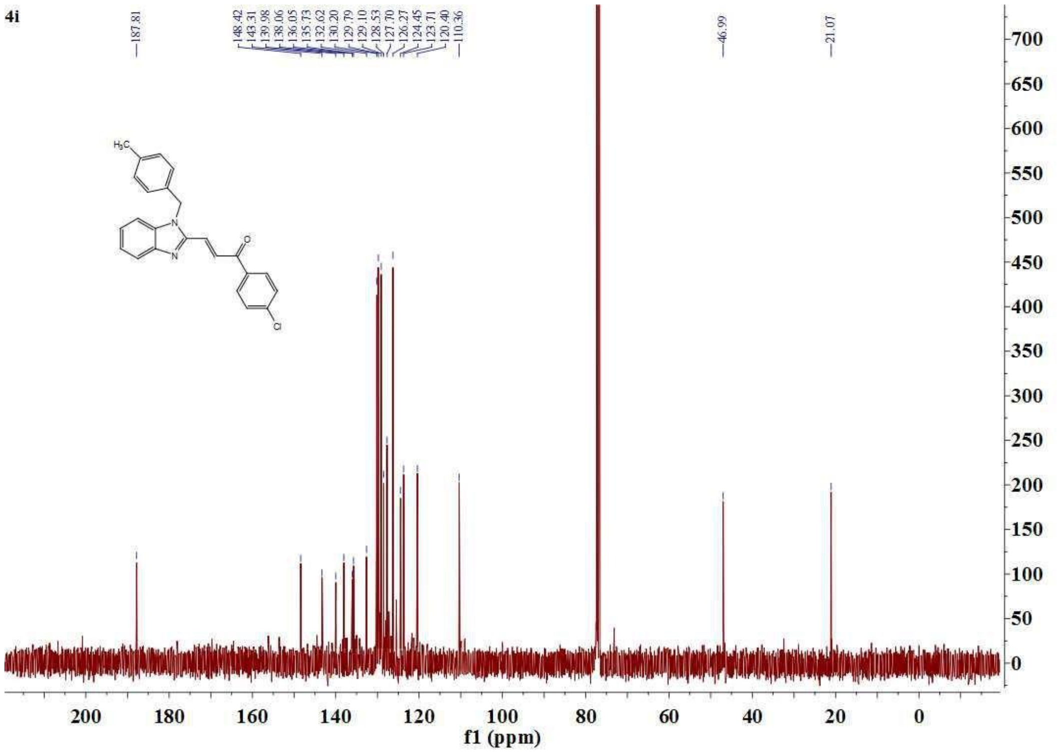

^{13}C NMR (101 MHz, $CDCl_3$) of compound **4i**

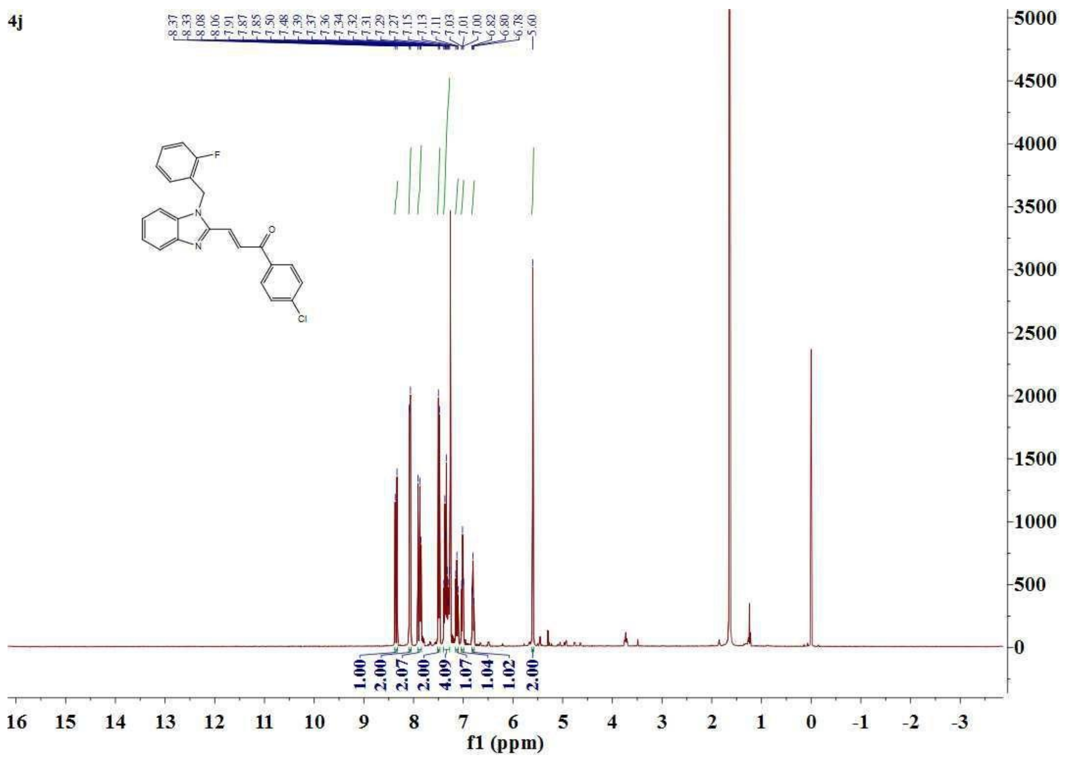

^{1}H NMR (400 MHz, $CDCl_3$) of compound **4j**

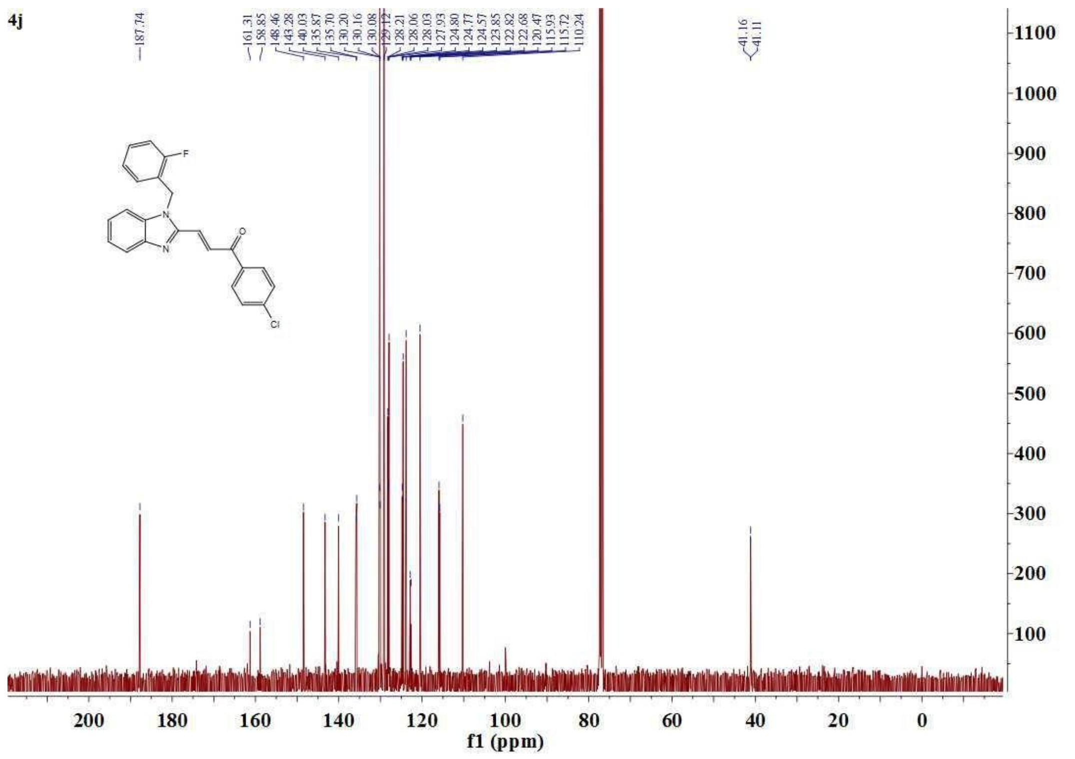

^{13}C NMR (101 MHz, $CDCl_3$) of compound **4j**

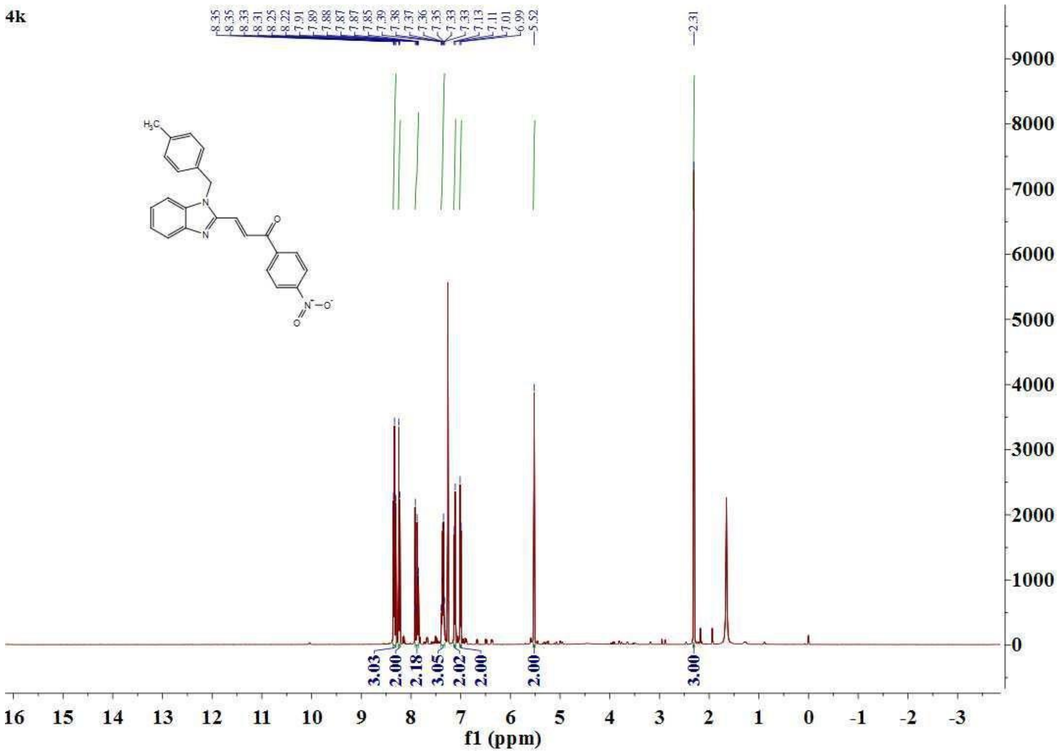

^{1}H NMR (400 MHz, $CDCl_3$) of compound **4k**

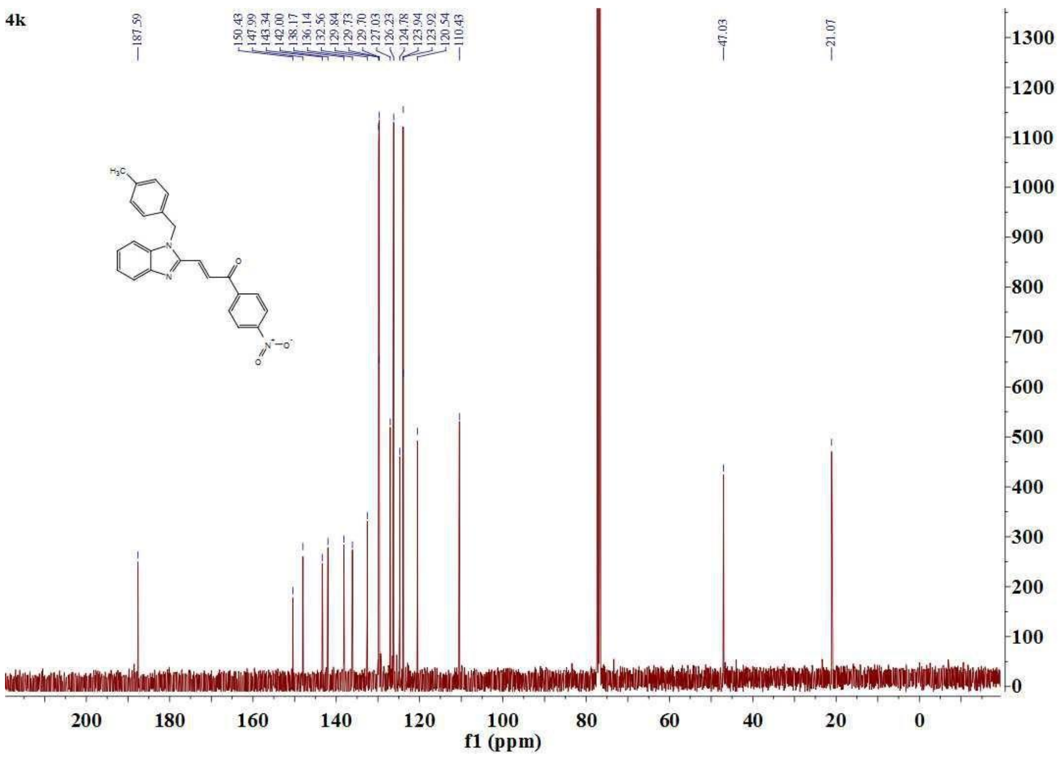

^{13}C NMR (101 MHz, $CDCl_3$) of compound **4k**

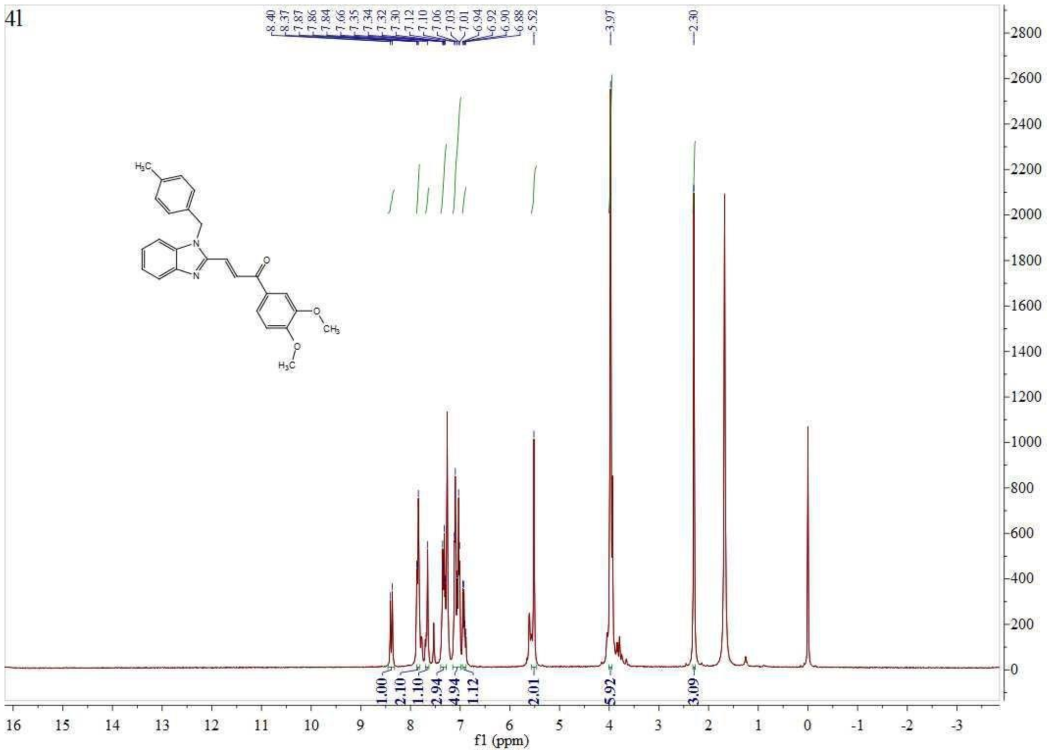

H NMR (400 MHz, CDCl 3) of compound **4l**

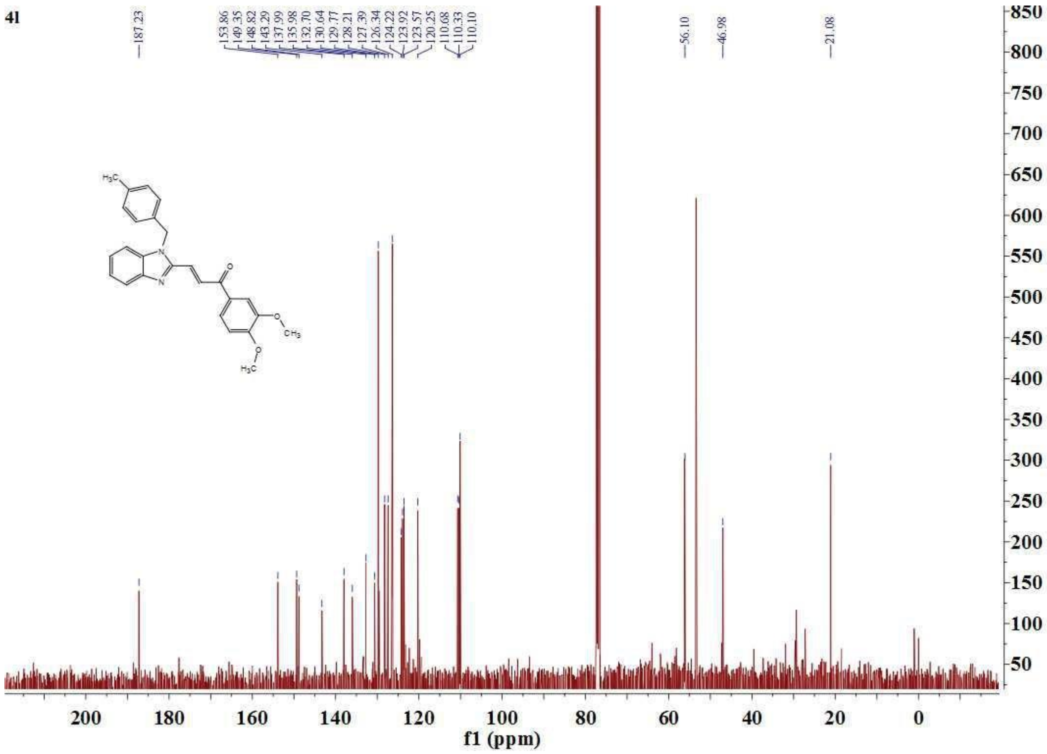

^{13}C NMR (101 MHz, CDCl 3) of compound **4l**

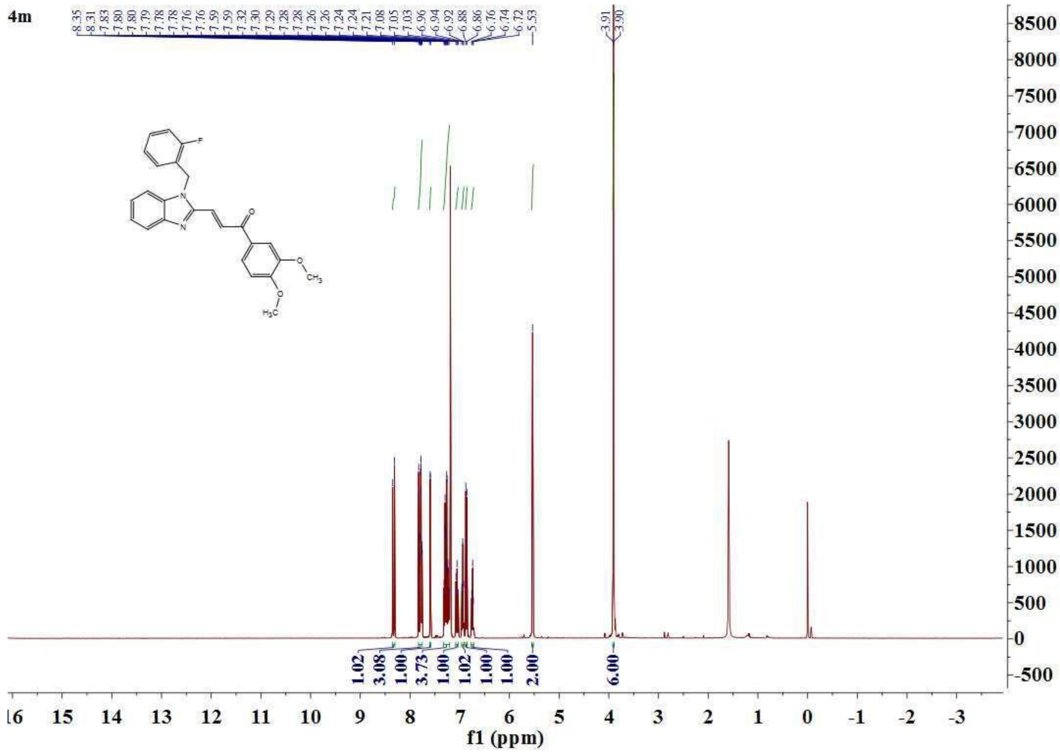

^{1}H NMR (400 MHz, $CDCl_3$) of compound **4m**

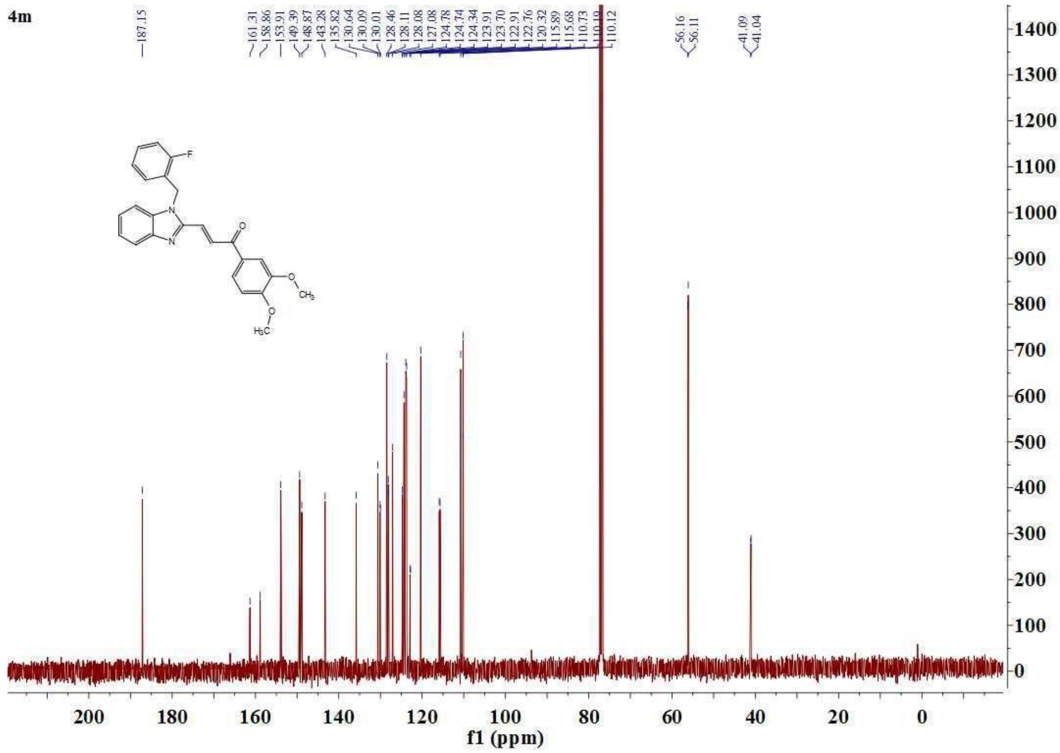

[13] C NMR (101 MHz, $CDCl_3$) of compound **4m**

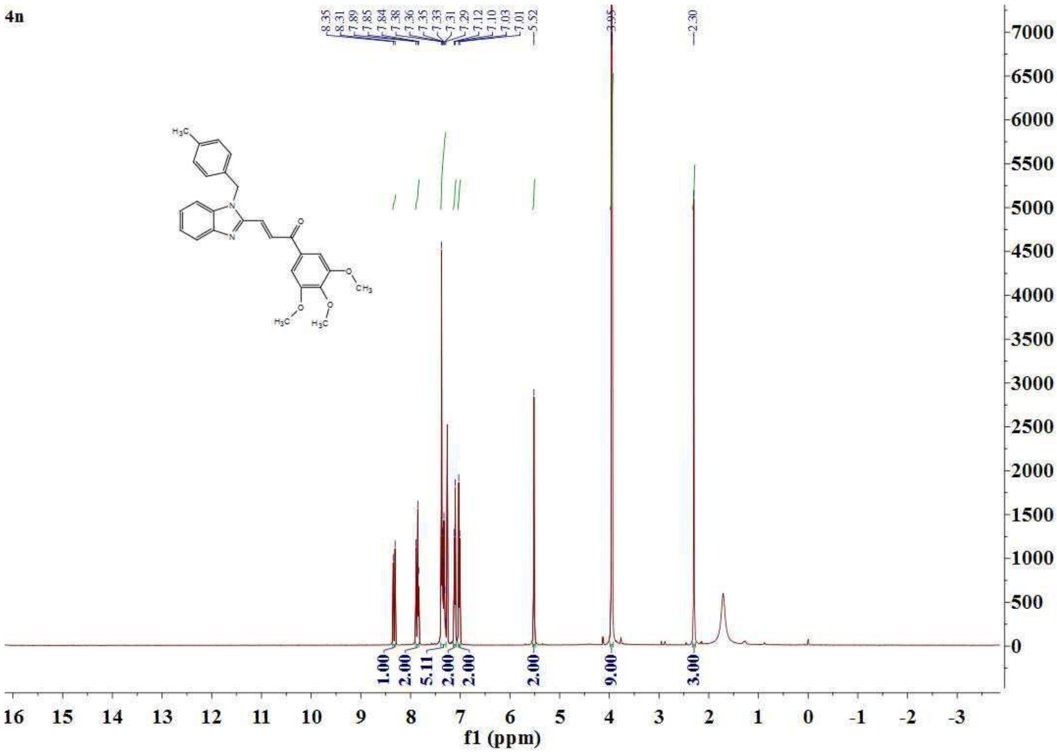

^{1}H NMR (400 MHz, $CDCl_3$) of compound **4n**

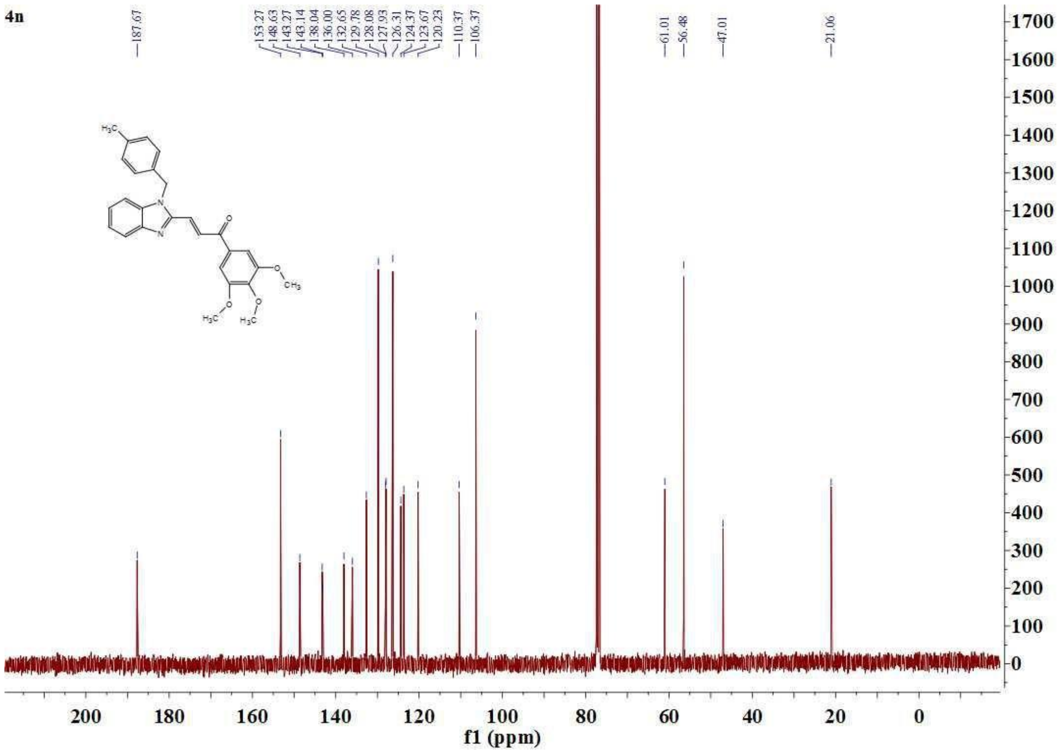

^{13}C NMR (101 MHz, $CDCl_3$) of compound **4n**

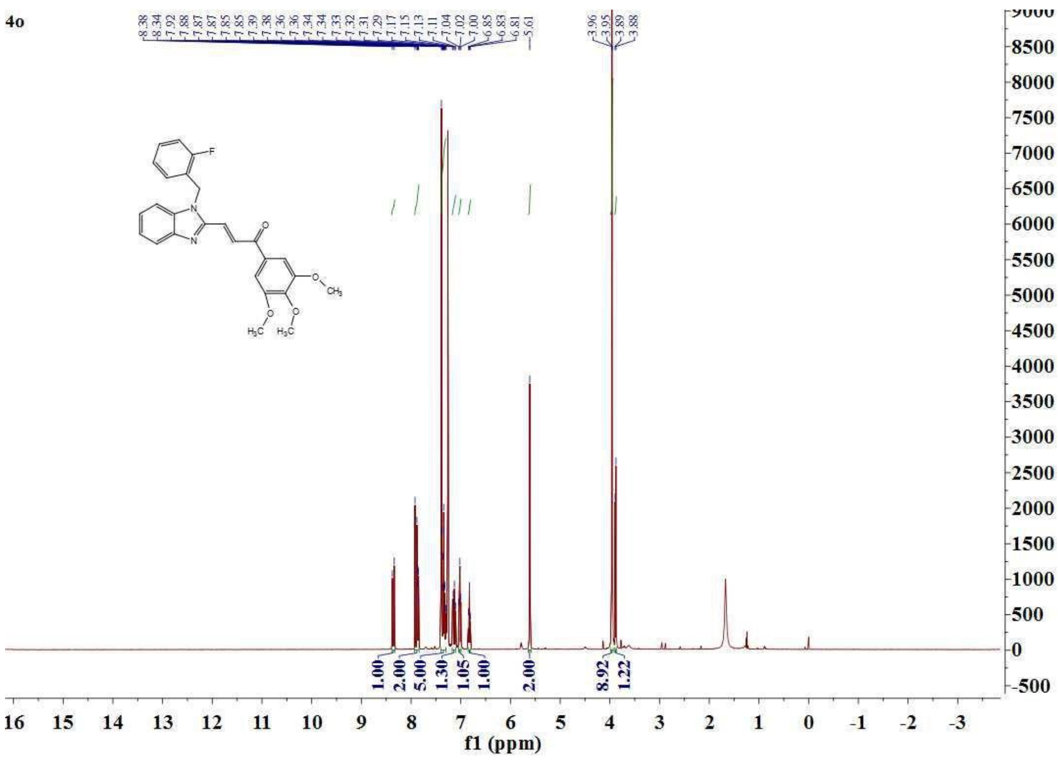

^{1}H NMR (400 MHz, CDCl $_{3}$) of compound **4o**

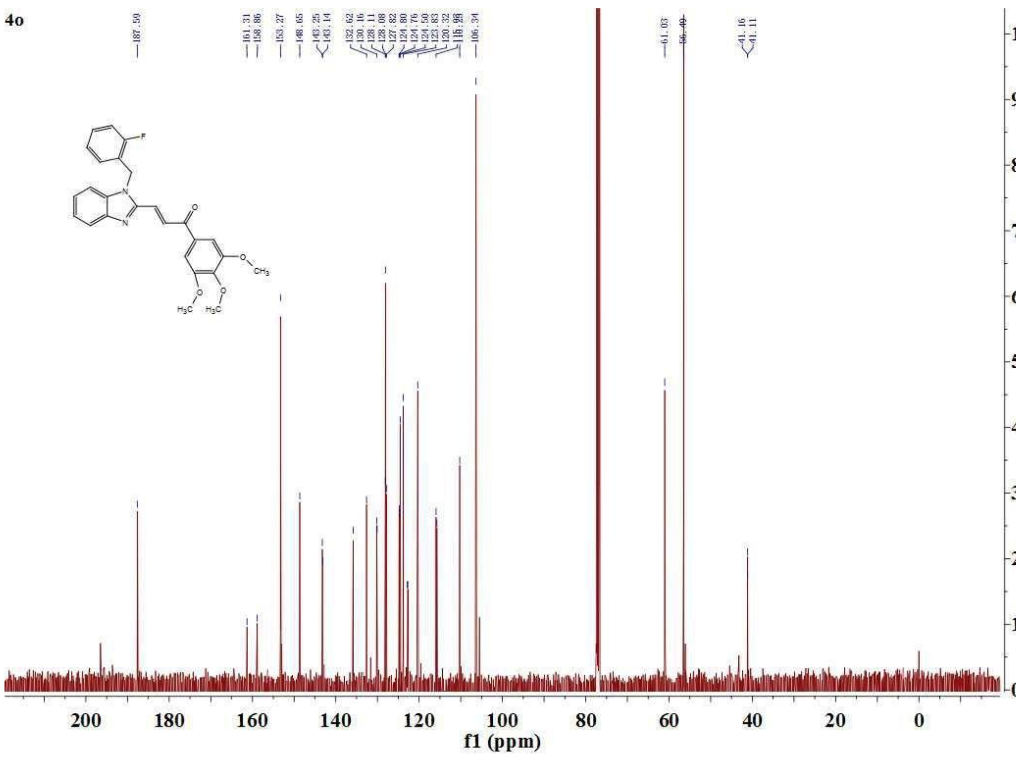

^{13}C NMR (101 MHz, CDCl $_{3}$) of compound **4o**

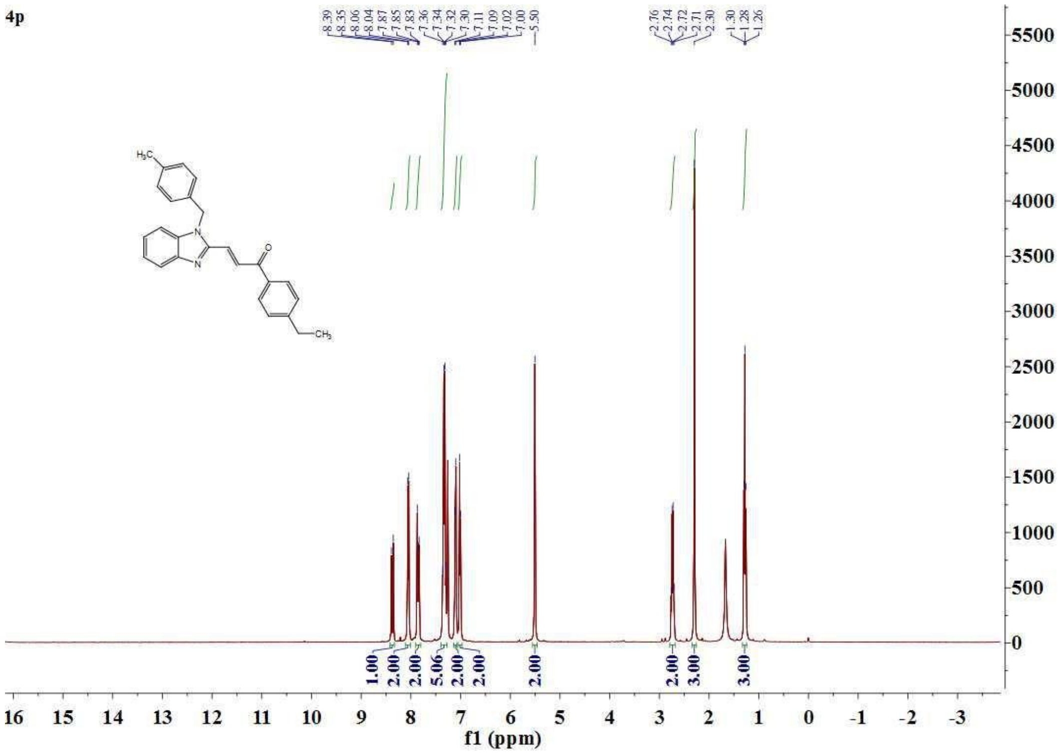

^{1}H NMR (400 MHz, $CDCl_3$) of compound **4p**

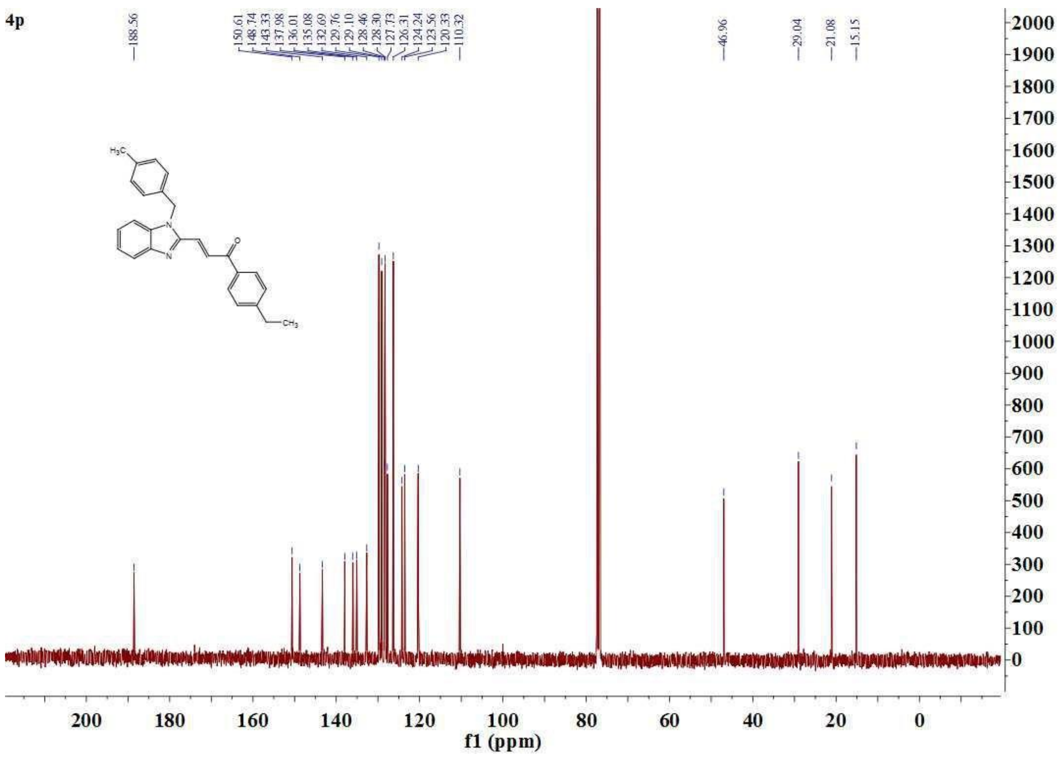

^{13}C NMR (101 MHz, $CDCl_3$) of compound **4p**

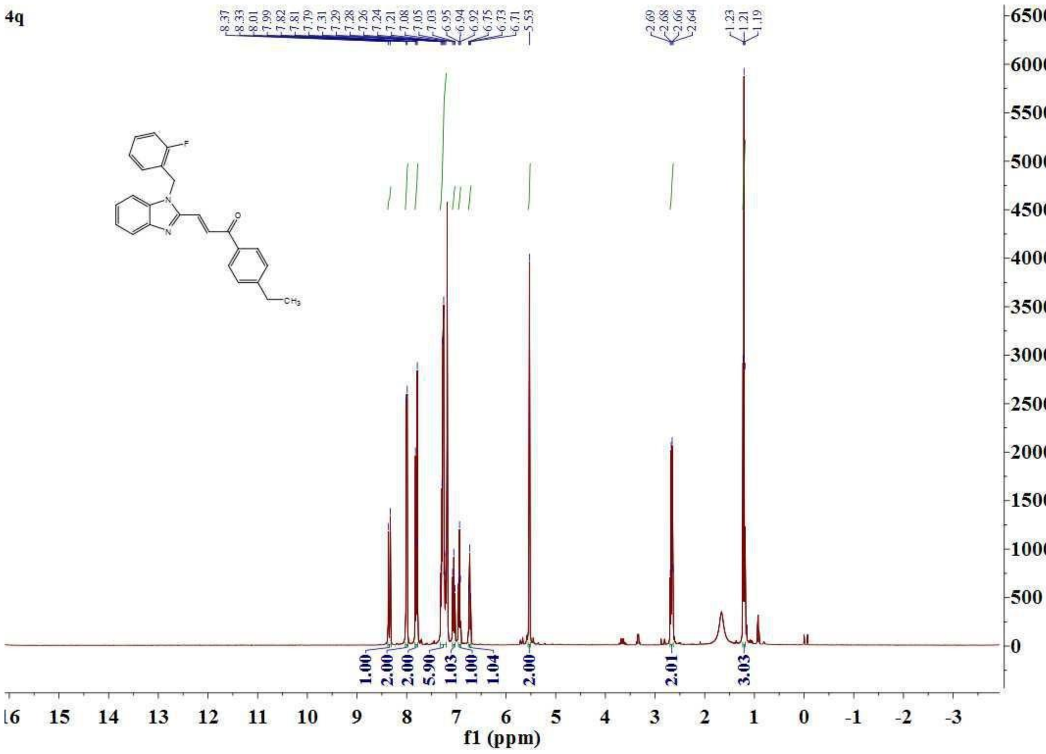

^{1}H NMR (400 MHz, CDCl $_3$) of compound **4q**

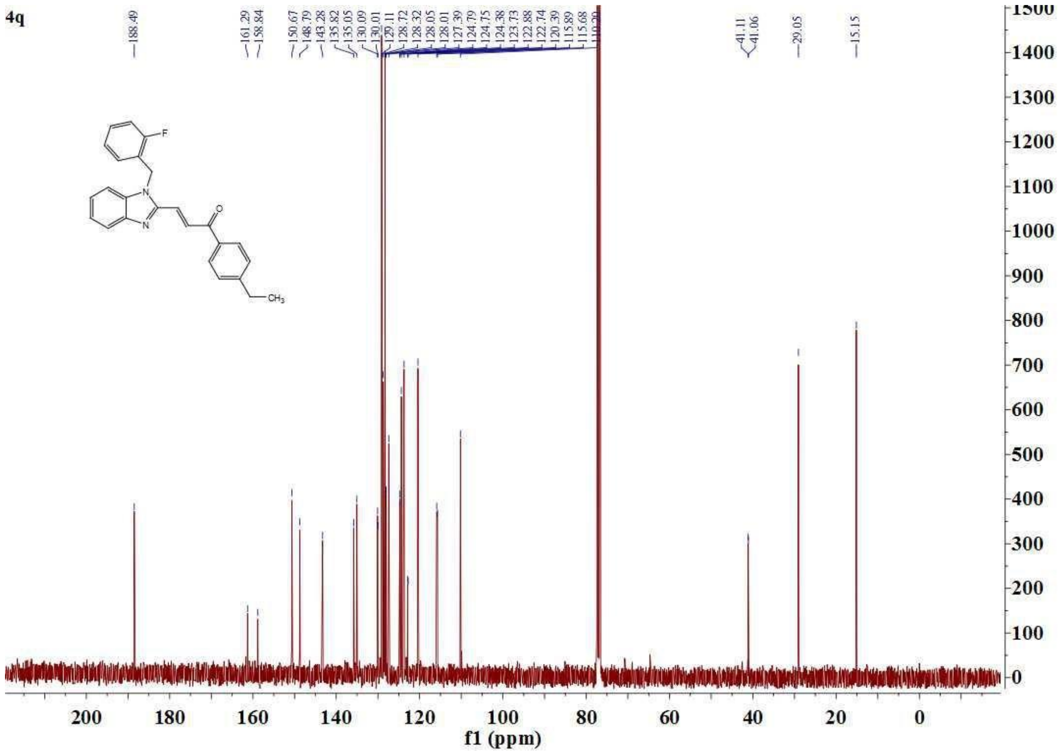

^{13}C NMR (101 MHz, CDCl $_3$) of compound **4q**

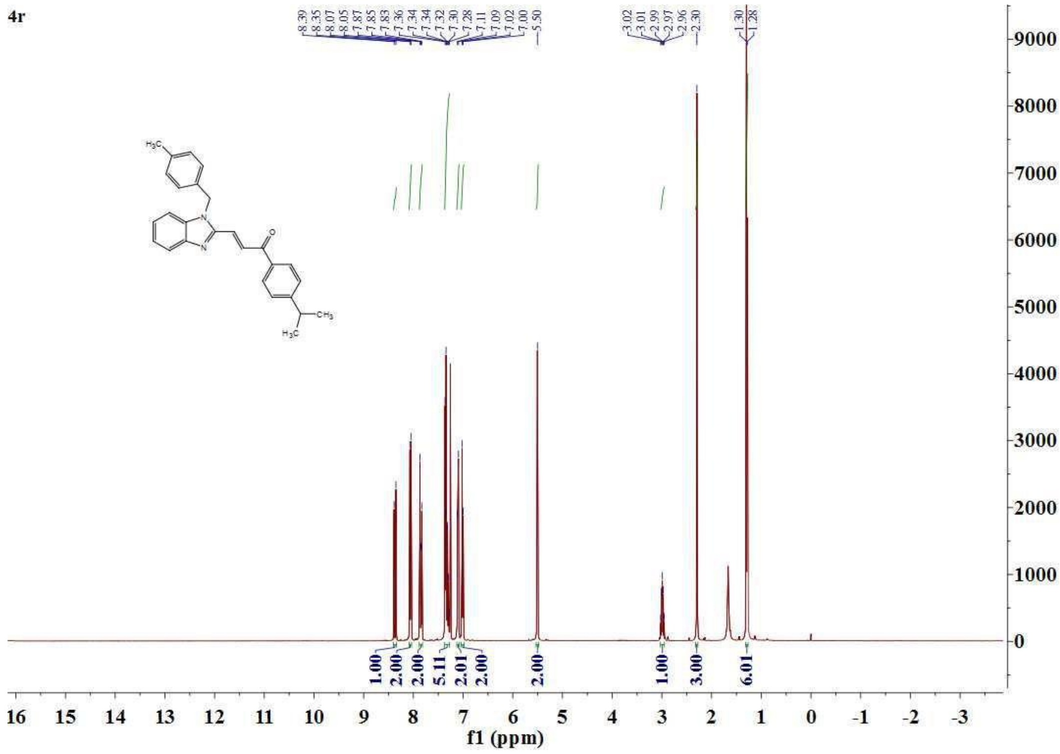

^{1}H NMR (400 MHz, $CDCl_3$) of compound **4r**

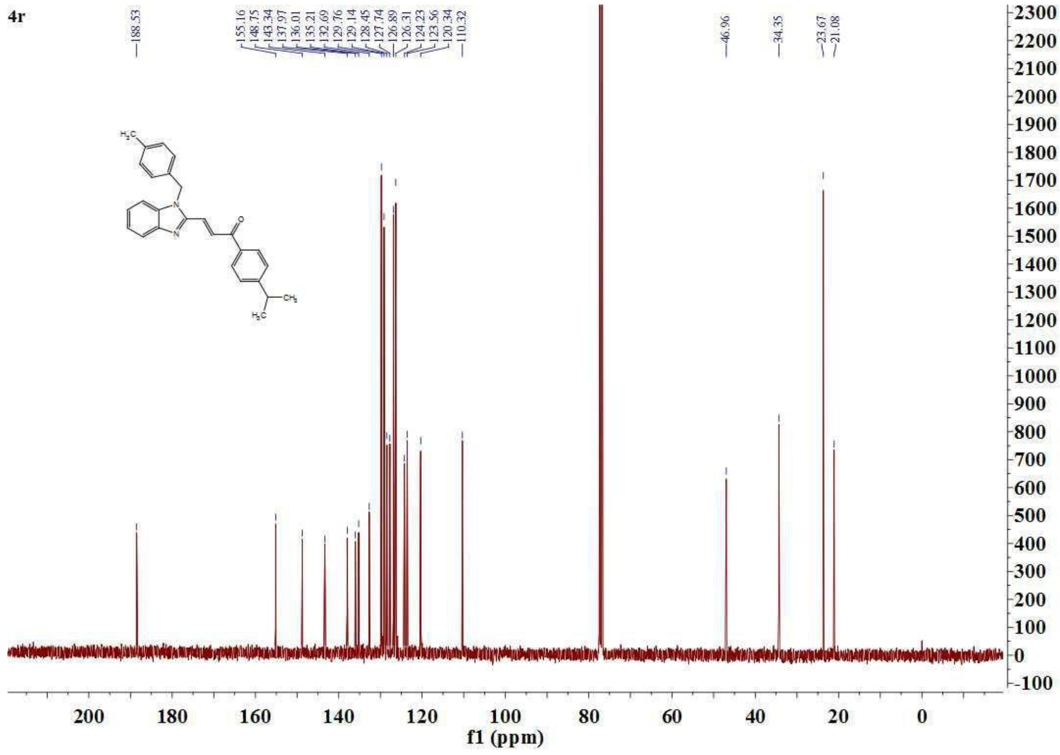

^{13}C NMR (101 MHz, $CDCl_3$) of compound **4r**

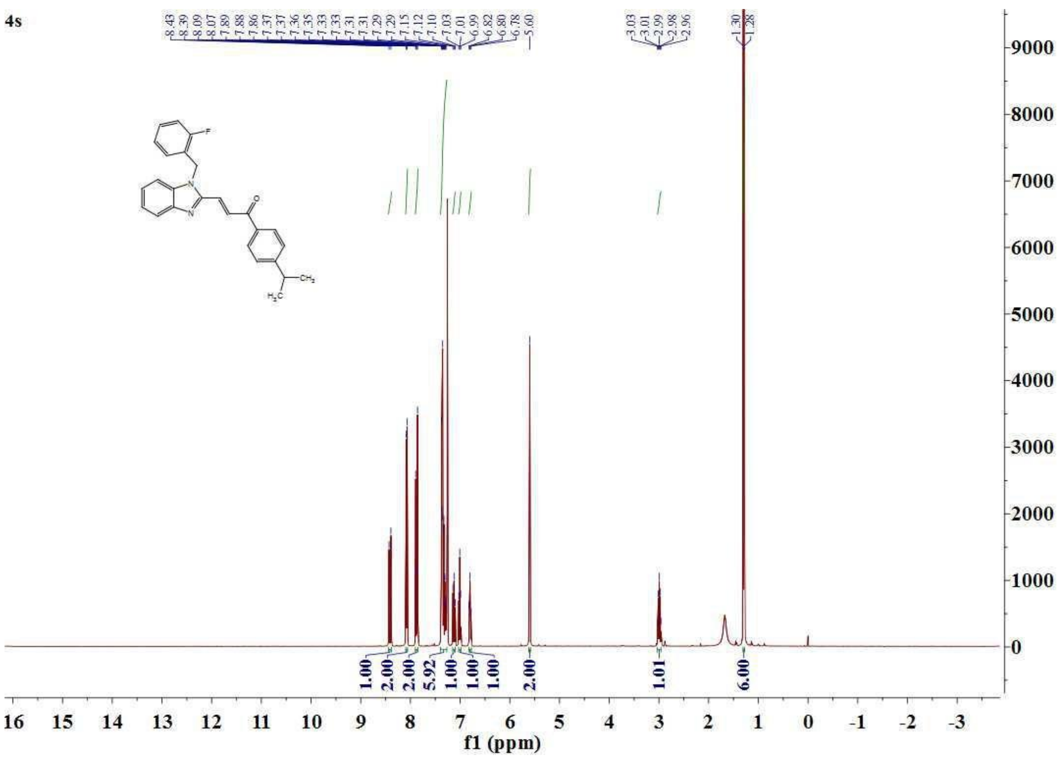

^{1}H NMR (400 MHz, $CDCl_3$) of compound **4s**

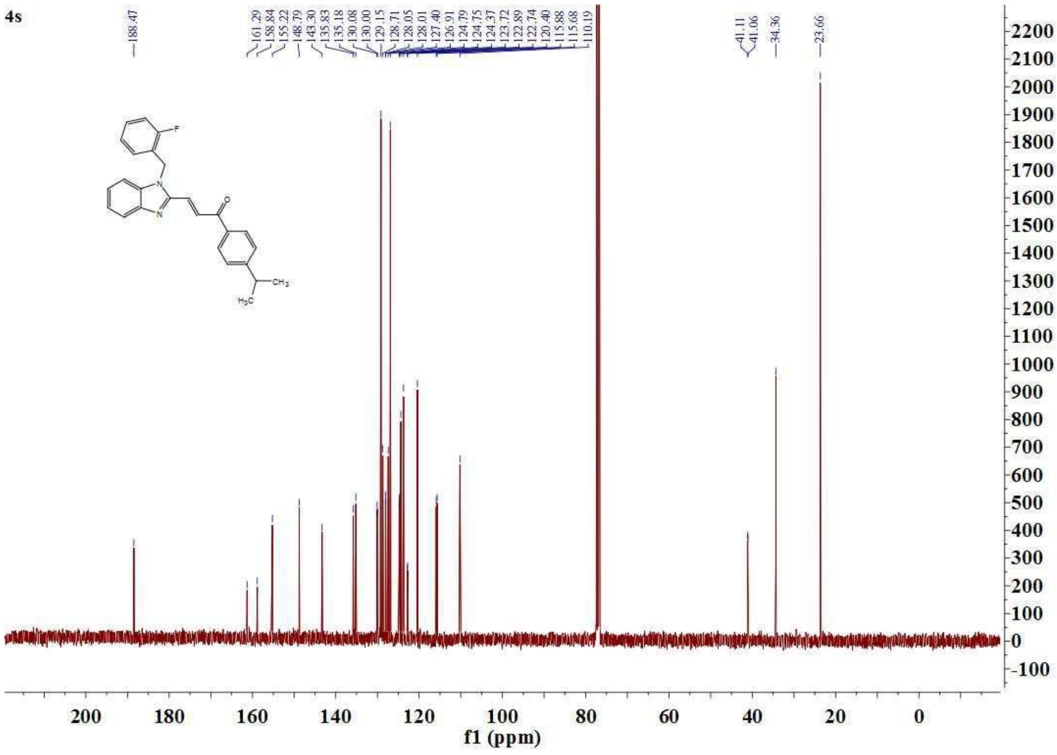

^{13}C NMR (101 MHz, $CDCl_3$) of compound **4s**

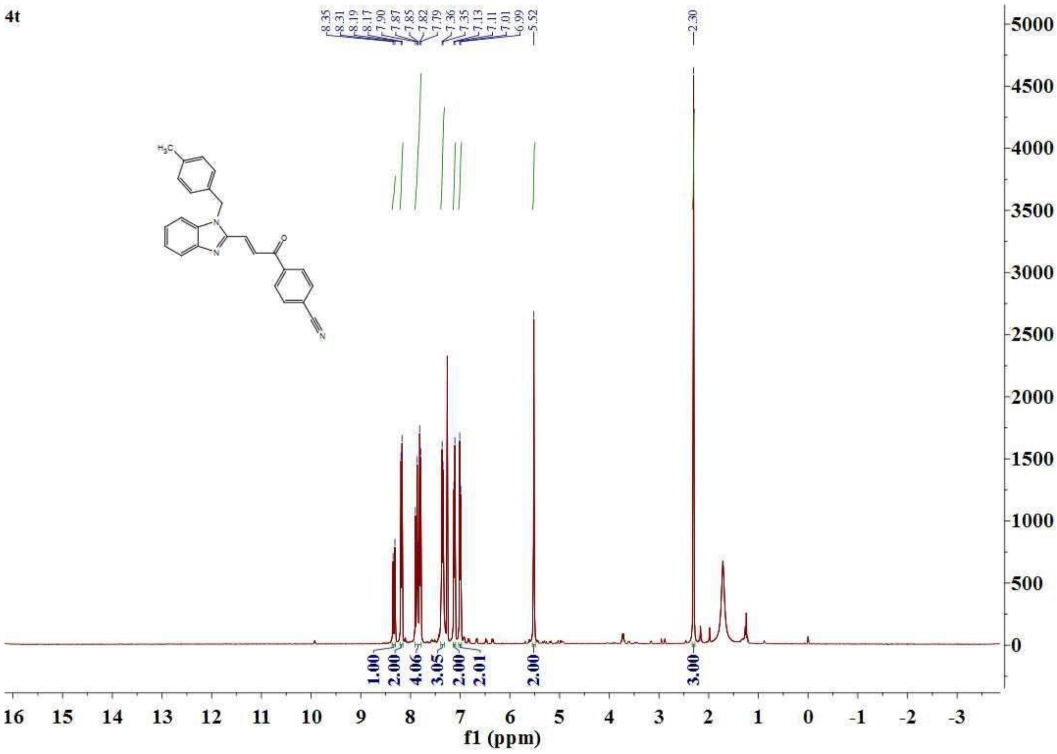

^{1}H NMR (400 MHz, CDCl 3) of compound **4t**

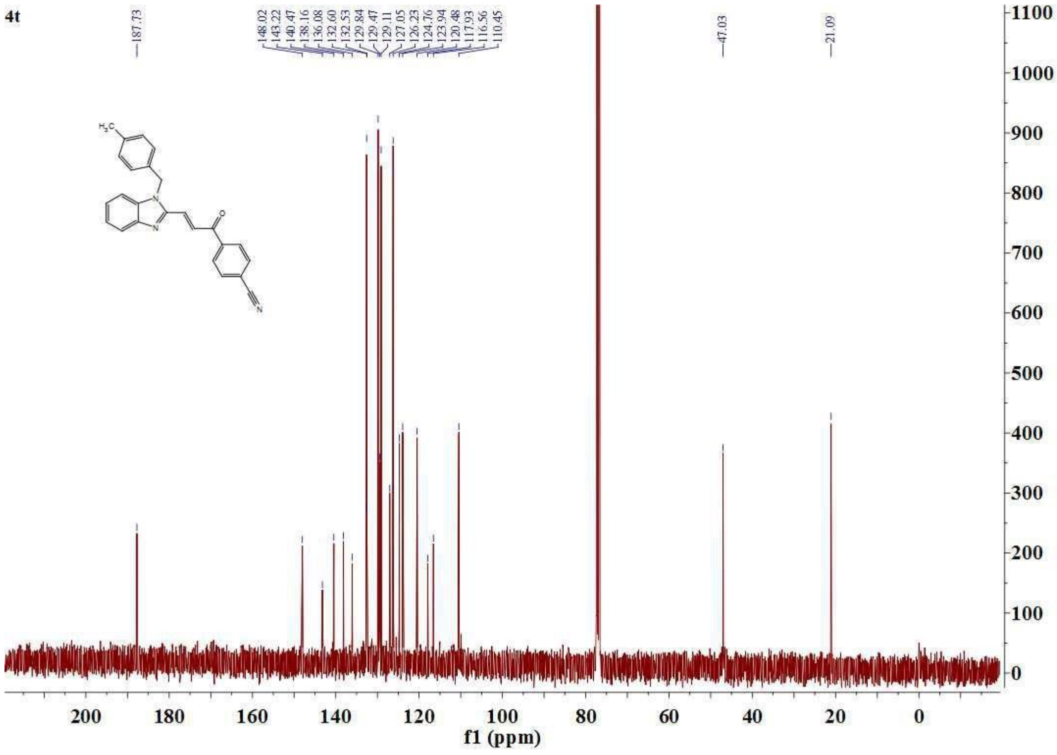

[13] C NMR (101 MHz, CDCl 3) of compound **4t**

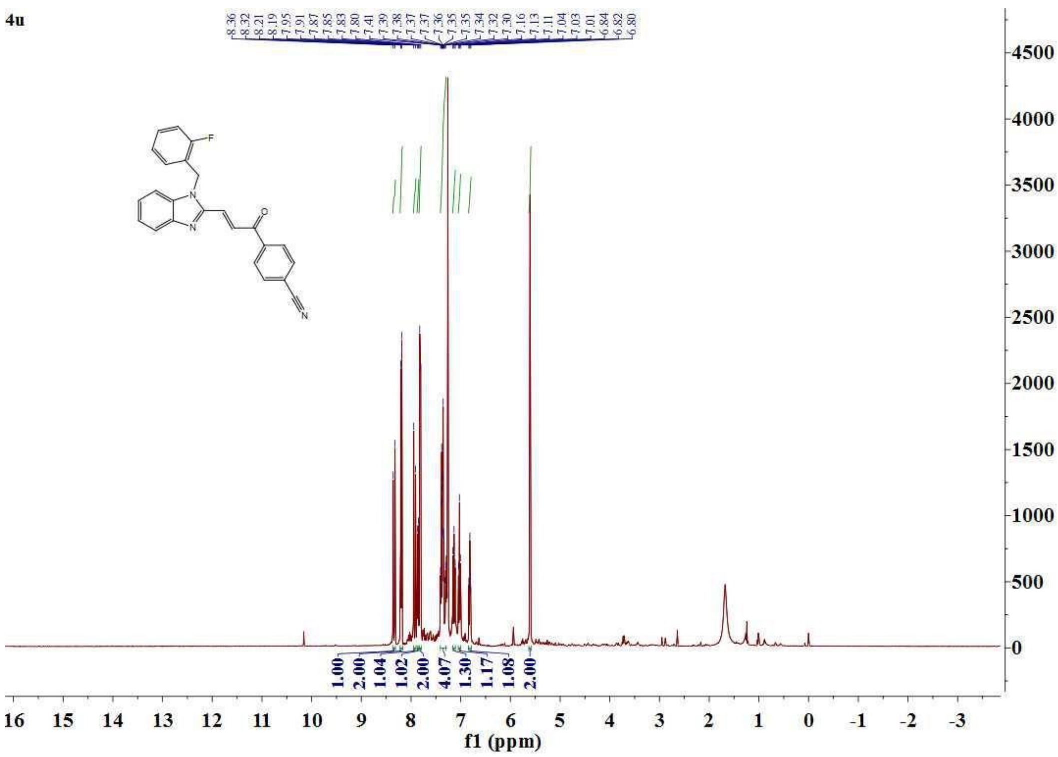

^{1}H NMR (400 MHz, $CDCl_3$) of compound **4u**

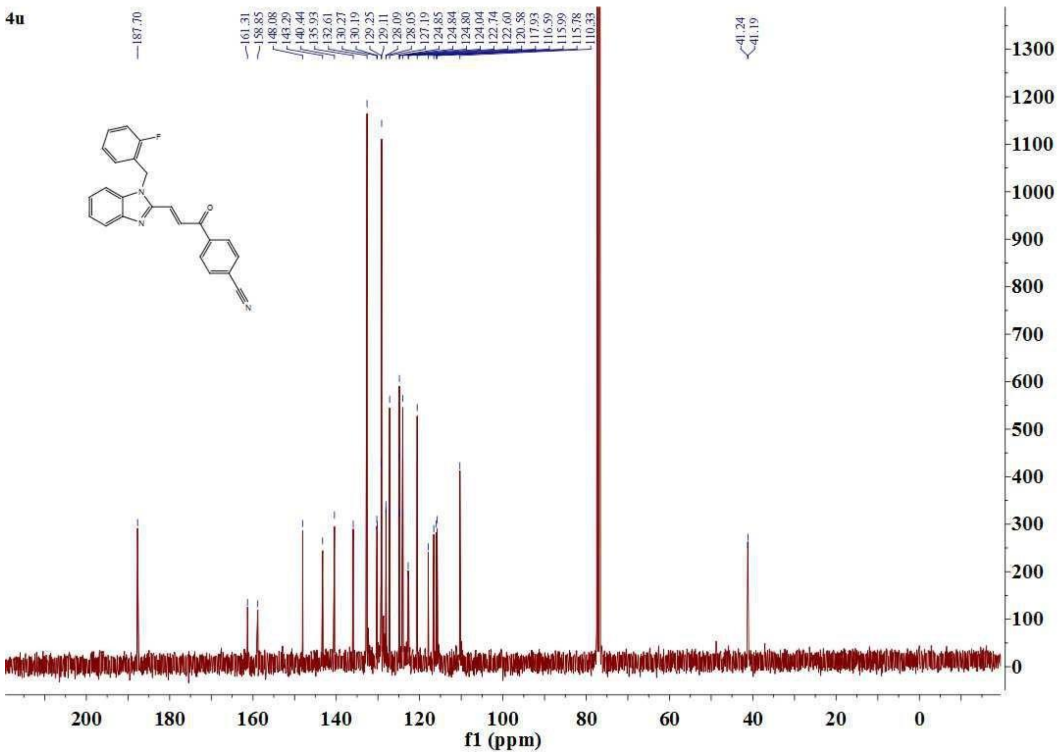

^{13}C NMR (101 MHz, $CDCl_3$) of compound **4u**

Supplementary Table 1: Total scores of 4n, 4d and original ligand of Topo II (1ZXM)

Spreadsheet

		Name	Structure	1: Total_Score	2: Crash	3: Polar	4: D_SCORE	5: PMF_SCORE	6: G_SCORE	7: CHEMSCORE	8: CSCORE	9: GLOBAL_CSCORE
1	☑	1zxm_ligand_000	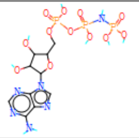	12.31	-2.42	8.96	-160.575	-76.006	-369.724	-22.601	4	3
2	☑	4n_000	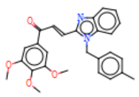	9.62	-3.74	1.28	-191.591	46.253	-311.829	-30.155	3	4
3	☑	4d_000	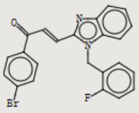	6.53	-0.63	1.12	-130.878	5.300	-180.310	-28.311	2	1
4	☑	334-18-9.mol_000	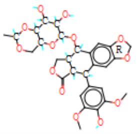	6.52	-7.83	2.52	-208.135	-0.424	-403.241	-34.455	1	3

Supplementary Figure 1: Docking model of 4n with Topo II protein (1ZXM)

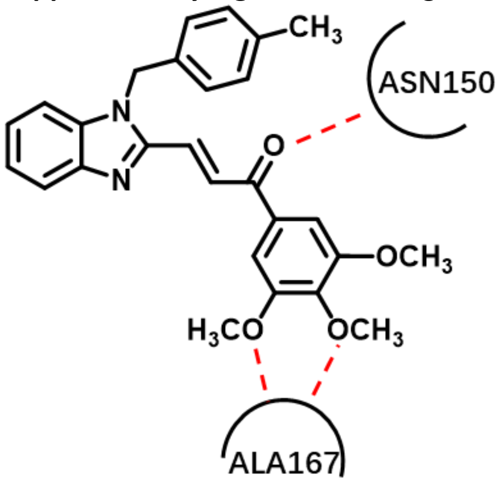

Supplementary Figure 2: Docking model of 4d with Topo II protein (1ZXM)

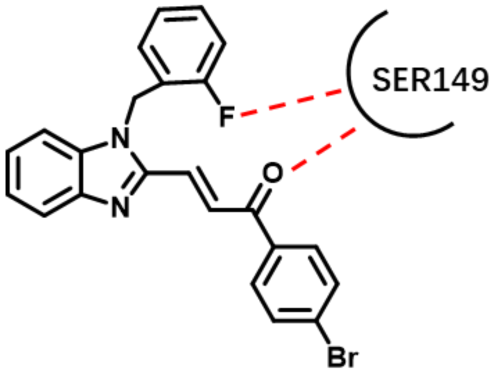

Supplementary Figure 3: Docking model of Topo II protein (1ZXM) with its original ligand

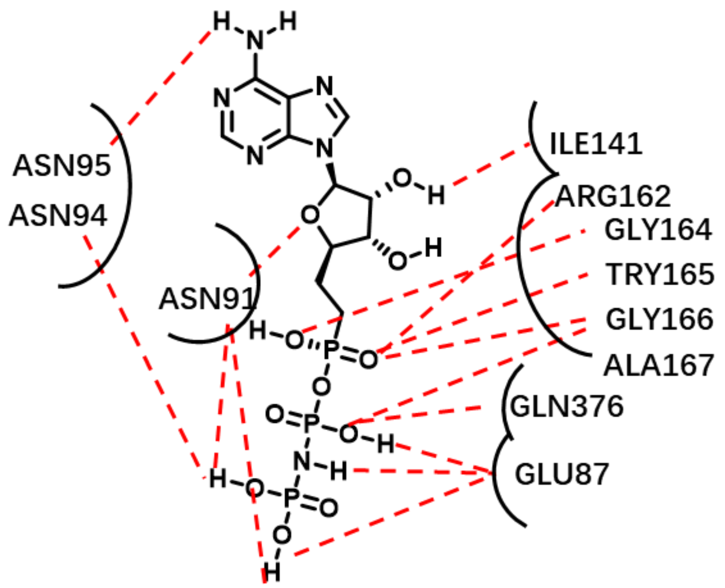

Supplementary Figure 4: Docking result of Topo II protein (1ZXM) with its original ligand

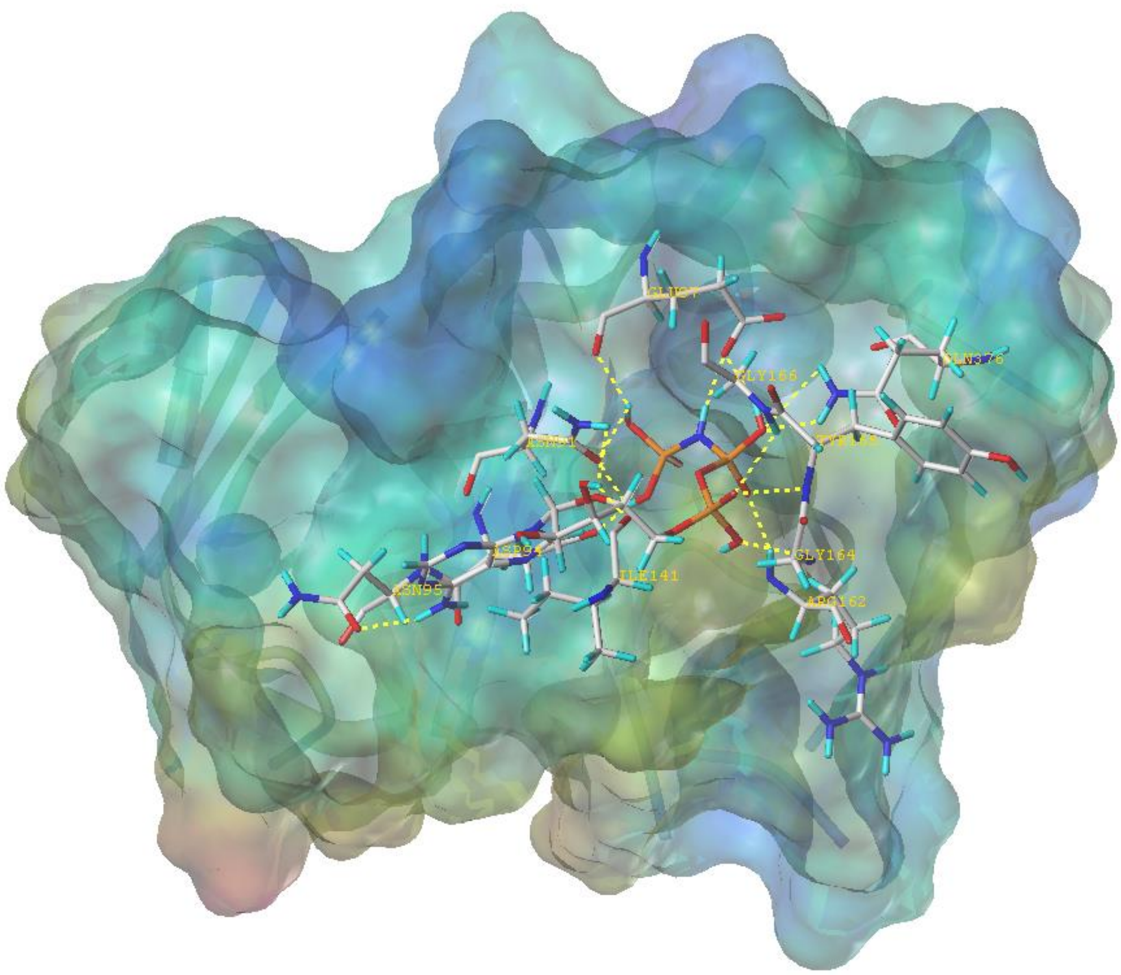

Supplement: Supplementary file 1 [file molecules-25-03180-s001.pdf]
